# Marker-Centered Evaluation of De Novo Assemblers Using Validated Microsatellite Loci in the Endangered Korean Fish *Pseudopungtungia nigra*

**DOI:** 10.3390/biology15201889

**Published:** 2026-10-20

**Authors:** Kang-Rae Kim, Keun-Sik Kim, In-Chul Bang

**Affiliations:** 1 Southeast Sea Fisheries Research Institute, National Institute of Fisheries Science, Namhae 52440, Republic of Korea; krkim89@korea.kr; 2 Department of Life Science, Soonchunhyang University, Asan 31538, Republic of Korea; 3 Restoration Research Team (Fishes/Amphibians & Reptile), Research Center for Endangered Species, National Institute of Ecology, Yeongyang 36531, Republic of Korea; kskim@nie.re.kr

**Keywords:** microsatellite, de novo assembly, assembler benchmarking, SPAdes, pseudo-reference

## Abstract

The recovery of experimentally validated microsatellite loci from short-read assemblies may provide an application-specific measure of assembly quality that complements conventional genome-wide statistics. In this study, four de novo assemblers—SPAdes, ABySS, SOAPdenovo2, and MaSuRCA—were evaluated using genomic reads from two individuals of the endangered Korean freshwater fish *Pseudopungtungia nigra*. Assembler performance was assessed based on assembly contiguity, BUSCO completeness, computational requirements, and the recovery of 20 PCR-validated microsatellite loci and their flanking regions. SPAdes produced the most contiguous assemblies and the highest BUSCO completeness among the tested assemblers, with N50 values of 5.5–7.0 kb and BUSCO completeness of 30.2–35.2%, and recovered all 20 loci and their flanking sequences at high confidence in both individuals. Marker recovery by ABySS, SOAPdenovo2, and MaSuRCA varied according to k-mer size and sample, with high-confidence recovery decreasing at larger k-mer sizes in several configurations and reaching 9 of 20 loci in the least favorable condition. SPAdes used 32.1–34.2 GB of peak memory, compared with up to 135.0 GB in the most memory-intensive SOAPdenovo2 runs. Reference-guided comparisons retained substantially greater total aligned sequence with the two *Pseudorasbora* references than with the more divergent *Cyprinus carpio* reference, although the mean identity of the smaller retained *C. carpio* alignment set was slightly higher. All 20 validated marker sequences produced high-confidence provisional placements in both *Pseudorasbora* reference genomes. As an exploratory downstream application, the SUC6832-derived pseudo-reference enabled joint genotyping of the two samples and yielded 2,012,643 filtered candidate variants across 25 guide-assigned scaffolds. These results indicate that SPAdes provided the most favorable balance of validated marker recovery, relative assembly quality, and memory efficiency among the tested configurations, although all assemblies remained fragmented at the genome-wide level. Marker-centered benchmarking can therefore complement conventional assembly statistics when short-read assemblies are generated primarily for microsatellite development, while pseudo-reference-based genomic analyses should remain provisional because of assembly fragmentation, reference bias, and limited biological sampling.

## Simple Summary

This study compared four de novo genome assemblers using 20 PCR-validated microsatellite loci from the endangered Korean fish *Pseudopungtungia nigra*. Under the tested conditions, SPAdes recovered all validated loci and their flanking regions in both individuals and used less peak memory than the most memory-intensive alternative configurations. Reference-guided organization of the SPAdes assembly also enabled provisional chromosome-scale marker localization and exploratory variant discovery. These results demonstrate the value of evaluating genome assemblies using experimentally validated loci in addition to conventional assembly statistics.

## 1. Introduction

The black shinner *Pseudopungtungia nigra* (Cyprinidae: Gobioninae) is a freshwater fish endemic to Korea and is legally protected as an endangered species. Its populations are fragmented across several river drainages and show signatures of both recent and historical bottlenecks, with previous microsatellite and mitochondrial DNA analyses revealing pronounced regional differentiation—most notably a genetically distinct Mangyeonggang population separated from the Geumgang system [1,2]. Effective conservation of such a species depends on molecular tools that can reliably quantify genetic diversity, delineate management units, and monitor populations over time [2].

Microsatellites, or simple sequence repeats (SSRs), remain among the most informative and cost-effective markers for the population genetics of non-model and threatened fishes, owing to their codominant inheritance, high polymorphism, reproducibility, and amenability to standardized genotyping [3,4,5]. Because they can be scored consistently across laboratories and time points, they continue to be widely used for parentage, connectivity, and diversity assessments even as sequencing-based approaches expand [4]. Constructing a validated and reproducible SSR panel, however, requires accurate reconstruction not only of the repeat tract itself but also of sufficiently long and conserved flanking regions for locus-specific primer design [6]. Short reads may not span the complete repeat and both flanking regions, while sequencing and assembly errors can further obscure genuine variation around SSR loci [6,7].

For endangered species that lack a chromosome-level reference genome, SSR markers are now most commonly discovered from high-throughput short-read data by mining de novo assemblies, an approach that has largely replaced traditional enrichment cloning across both plants and animals [8]. This strategy makes assembly quality an important determinant of marker recovery, because assembler choice and k-mer parameters can substantially affect assembly contiguity, completeness, and computational requirements [9]. Community benchmarking efforts such as Assemblathon 1 and Assemblathon 2 have repeatedly shown that different assemblers can produce markedly different reconstructions of the same vertebrate genome, and that no single tool is uniformly optimal across datasets [9,10,11].

Assembly quality is conventionally summarized using global statistics—total length, N50, and the recovery of conserved single-copy orthologs (BUSCO)—computed with standardized tools such as QUAST and BUSCO [12,13]. These metrics are essential for describing overall contiguity and completeness, but they are genome-wide averages, and whether they predict the recovery of specific, biologically validated loci in a low-coverage vertebrate assembly remains largely untested [14]. Because locus-level recovery ultimately determines whether a usable marker can be developed, benchmarking based on validated markers can provide an application-specific assessment that complements genome-wide measures of assembly contiguity and completeness [15].

Beyond marker development, conservation genetics is increasingly shifting from small marker panels toward genome-scale data, which offer greater power to estimate diversity, infer demographic history, and identify adaptive variation [16,17,18]. Whole-genome and reduced-representation sequencing approaches—including RAD sequencing and genotyping-by-sequencing—have been applied successfully to non-model and threatened fishes, although the availability and quality of a reference genome can substantially influence read mapping, variant discovery, and downstream inference [17,19,20,21]. When a species-specific reference is unavailable, reference-guided scaffolding against related species and the construction of a sample-derived pseudo-reference can extend fragmented short-read assemblies into provisional genome-wide frameworks for placing markers at the chromosome scale and surveying variation [18]. The reliability of these analyses may be affected by phylogenetic distance, reference architecture, and reference bias. Reference-guided rearrangements should therefore be treated as alignment-derived candidates, whereas variants called against a sample-derived pseudo-reference should be regarded as provisional until independently validated [17,18].

Despite the widespread use of de novo assemblies for microsatellite development, few studies have evaluated assembler performance using loci that have already been experimentally validated. In particular, it remains unclear whether assemblers that perform well according to conventional genome-wide statistics also consistently reconstruct complete SSR loci and their primer-binding flanking regions across different parameter settings and biological individuals. A distinguishing feature of the present study is therefore the use of PCR-validated SSR loci as application-specific benchmarking targets for de novo assembly evaluation. Conventional metrics such as N50 and BUSCO provide important measures of genome-wide contiguity and conserved gene-space completeness, respectively, but they do not directly assess whether experimentally functional SSR loci and the flanking sequences required for primer design have been reconstructed. Recovery of PCR-validated SSR loci therefore provides a complementary, locus-level measure of assembly utility rather than a replacement for conventional assembly statistics. Accordingly, this study compared multiple de novo assemblers and k-mer conditions using a defined set of validated SSR markers alongside conventional assembly metrics and computational requirements.

Here, using the endangered Korean cyprinid *P. nigra* as a model, we primarily evaluated de novo assemblers using validated SSR loci and subsequently examined the utility of the assembly showing the most favorable performance under the tested conditions for reference-guided marker localization and exploratory variant analysis. Specifically, we (i) developed a set of PCR-validated SSR markers from short-read de novo assemblies of two individuals from different drainages; (ii) evaluated four widely used de novo assemblers—SPAdes, ABySS, SOAPdenovo2, and MaSuRCA—across multiple k-mer settings, using high-confidence recovery of the validated markers and their flanking regions as the primary benchmark alongside assembly quality, completeness, and computational cost; (iii) examined contig- and chromosome-scale reproducibility between the two individuals through reference-guided scaffolding against two relatively closely related gobionine references (*Pseudorasbora elongata* and *P. parva*) and the more distantly related *Cyprinus carpio* reference; and (iv) localized the validated markers across these reference genomes and assessed the extent to which a sample-derived pseudo-reference could support provisional genome-wide variant analysis. Together, these analyses aim to establish a practical framework for SSR marker development and preliminary genome-wide variant exploration in non-model and threatened species for which a high-quality species-specific reference genome is not yet available.

## 2. Materials and Methods

### 2.1. De Novo Assembly for Development of Validated SSR Markers

Two individuals of *P. nigra* were used for microsatellite marker discovery: SUC6832, collected from Yudeungcheon Stream in the Geumgang River system, and SUC8040, collected from the Mangyeonggang River. These individuals represented two geographically and genetically differentiated drainage systems previously identified in population-genetic studies of *P. nigra* [1,2] and were used as independent biological datasets to evaluate the reproducibility of assembler-dependent marker recovery. The present study was designed as a technical assembler benchmark rather than a population-level comparison; therefore, the two individuals were analyzed independently and were not treated as replicate populations. Genomic DNA was extracted from pelvic fin tissue using a Genomic DNA Prep Kit (BioFact, Seoul, Republic of Korea) according to the manufacturer’s instructions. DNA concentration and purity were assessed using a NanoDrop ND-1000 spectrophotometer (Thermo Scientific, Wilmington, DE, USA).

Paired-end genomic libraries were sequenced on an Illumina MiSeq platform using a MiSeq Reagent Kit v3 (Illumina, Inc., San Diego, CA, USA, 600 cycles), generating approximately 2 × 300-bp paired-end reads. Sequencing of SUC6832 produced 36,468,638 reads comprising 10,977,060,038 bp, whereas sequencing of SUC8040 generated 40,417,926 reads comprising 12,165,795,726 bp.

For SUC6832, read 1 and read 2 each contained 18,234,319 reads, with a GC content of 39%. The Q20 values were 93% and 86%, and the Q30 values were 88% and 76% for read 1 and read 2, respectively. For SUC8040, read 1 and read 2 each contained 20,208,963 reads, with a GC content of 39%. The corresponding Q20 values were 94% and 90%, and the Q30 values were 89% and 81%, respectively. The raw reads were subsequently subjected to quality filtering and de novo assembly.

Paired-end reads from samples SUC6832 and SUC8040 were independently assembled de novo using CLC Genomics Workbench v12.1 (QIAGEN Digital Insights, Aarhus, Denmark) with default parameters. Automatic word size and bubble size selection were applied, with a minimum contig length of 200 bp, automatic paired-read distance detection, scaffolding enabled, and read mapping back to contigs enabled. Default mapping parameters were used, including mismatch cost 2, insertion cost 3, deletion cost 3, length fraction 0.5, and similarity fraction 0.8.

Contigs shared between the SUC6832 and SUC8040 assemblies were identified and retained as common contigs. These common contigs were screened for microsatellite loci, yielding 936 shared SSR candidates. Among these, 50 loci were randomly selected from candidates containing dinucleotide repeats with at least six repeat units and an expected PCR amplicon size of ≤600 bp. Primer pairs were manually designed using conserved flanking sequences.

PCR amplification was performed in a total reaction volume of 20 μL containing 2 μL of AccuPower PCR PreMix (Bioneer, Daejeon, Republic of Korea), 0.8 μM each of fluorescently labeled forward (FAM or HEX) and reverse primers, and 100 ng of genomic DNA, with nuclease-free water added to the final volume. The thermal cycling conditions consisted of an initial denaturation at 94 °C for 3 min, followed by 34 cycles of denaturation at 94 °C for 30 s, annealing at 58 °C for 30 s, and extension at 72 °C for 30 s, with a final extension at 72 °C for 7 min. Amplified fragments were analyzed by capillary electrophoresis using an ABI 3730xl Genetic Analyzer (Applied Biosystems, Foster City, CA, USA). Allele sizes were determined using GeneScan 500 LIZ (Life Technologies Ltd., Warrington, UK) as an internal size standard and Peak Scanner software v1.0.

Of the 50 candidate loci, 20 produced reliable amplification and interpretable polymorphic allele profiles, with polymorphism information content (PIC) values > 0.3, as calculated using CERVUS v3.0. These loci constituted the final validated SSR marker panel, corresponding to an experimental validation success rate of 40% (20/50). The final panel was established through experimental validation rather than selection based on subsequent recovery performance in the assemblers evaluated in this study.

### 2.2. Sequence Data and Multi-Assembler Genome Assembly

Assemblies were independently generated using SPAdes v4.0.0 [11], ABySS v2.0.2 [22], SOAPdenovo2 r242 [23], and MaSuRCA v3.4.2 [24] with each program configured to use up to 25 threads or processes where supported. SPAdes v4.0.0 [11] was run once using a combined multi-k configuration of 21, 33, 55, 77, 99, and 127, whereas ABySS v2.0.2 [22], SOAPdenovo2 r242 [23], and MaSuRCA v3.4.2 [24] were evaluated in separate runs at k-mer sizes of 41, 71, and 127. SOAPdenovo2 r242 [23] was run with the -R option, a maximum read length of 301 bp, and an average insert size of 500 bp. MaSuRCA v3.4.2 [24] was run with USE_LINKING_MATES = 1, LIMIT_JUMP_COVERAGE = 300, KMER_COUNT_THRESHOLD = 1, JF_SIZE = 200000000, and cgwErrorRate = 0.15.

Assembly contiguity, BUSCO completeness, and computational requirements were evaluated alongside marker recovery to compare the overall performance of the tested configurations. QUAST v5.3.0 [13] was used to calculate total assembly length, sequence number, largest scaffold, N50, L50, and GC content. Genome completeness was assessed using BUSCO v6.1.0 [12] in genome mode with the actinopterygii_odb12 dataset and Miniprot v0.18-r281 [25].

Because the available sequencing depth was insufficient for a stable k-mer-based genome-size estimate, genome size was not inferred from the raw-read k-mer distribution. Instead, the amount of sequence data available for each individual was summarized descriptively as the ratio of total raw sequenced bases to the corresponding SPAdes assembly span. This yielded approximately 13.8× for SUC6832 (10.98 Gb/795.2 Mb) and 14.9× for SUC8040 (12.17 Gb/815.1 Mb). These values were used only as sequence-yield-to-assembly-span ratios and were not interpreted as independent estimates of true physical genome coverage.

To evaluate whether the relatively low BUSCO completeness of the *P. nigra* assemblies could reflect incompatibility with the selected lineage dataset rather than assembly fragmentation, the same BUSCO workflow and actinopterygii_odb12 dataset were additionally applied to the chromosome-level *Pseudorasbora parva* (GCF_024679245.1) and *P. elongata* (GCA_046055825.1) reference assemblies as lineage controls.

Wall-clock runtime, CPU utilization, and peak memory usage were recorded using GNU time and process-tree monitoring. Only completed assembly runs were included in comparisons of assembly quality, resource use, and marker recovery.

### 2.3. Assessment of Target Marker Sequence Recovery and Reproducibility

The primary analysis evaluated whether 20 target *P. nigra* marker sequences could be consistently recovered from assemblies generated using different assemblers, k-mer sizes, and biological samples. Each assembly was converted into a nucleotide BLAST database using makeblastdb v2.17.0+ [26], and the marker sequences were searched using BLASTn v2.17.0+ [26] with -max_target_seqs 5 and 16 threads.

For each marker, the alignment with the highest bitscore was retained as the primary hit. A marker was classified as detected when at least one BLAST hit was identified and as high-confidence when sequence identity was ≥95% and query coverage was ≥90%. Because high overall sequence similarity does not necessarily ensure preservation of functional primer-binding sites, the experimentally validated forward and reverse primer sequences were additionally evaluated separately within each marker sequence. Primer-binding regions were identified according to the known primer sequences and their positions within the corresponding marker sequence, and nucleotide identity and mismatches were recorded for each forward and reverse primer. Preservation of both primer-binding regions was used as an additional locus-level validation criterion.

Marker recovery rate was calculated as:

Marker recovery rate (%) = number of high-confidence recovered markers/20 × 100

Recovery rates were compared among assemblers, k-mer conditions, and samples SUC6832 and SUC8040 to identify assembly conditions that consistently reconstructed the complete marker set. The same procedure was applied to SSR marker sequences containing 100-bp flanking regions on both sides to evaluate their recovery across assembly conditions. In addition, the experimentally validated forward and reverse primer-binding sequences were examined independently to confirm nucleotide-level preservation of the primer annealing sites. Primer sequence, primer length, position within the marker sequence, percentage identity, and the number of mismatched nucleotides were recorded for each primer and are summarized in Supplementary Table S2.

The annotated SSR repeat tract in each of the 20 marker sequences was masked by replacing it with Ns using a custom script written in Python v3.11.3. Paired-end raw reads from samples SUC6832 and SUC8040 were mapped to the masked marker sequences using BWA-MEM v0.7.17-r1188 [27] with default parameters and 16 threads. Mapped reads were retained using SAMtools v1.23.1 [28] view with -F 4, sorted with SAMtools v1.23.1 [28] sort, and coverage breadth and depth were calculated using SAMtools v1.23.1 [28] depth with the -aa option. Markers with breadth coverage ≥90% were considered supported by the raw-read dataset.

### 2.4. Methods for Contig-Level Comparison of Marker-Recovering Assemblies

Contig-level comparisons were performed to describe the sequence correspondence retained between the SUC6832 and SUC8040 assemblies generated under matched assembler conditions. Because the short-read assemblies contained very large numbers of fragmented scaffolds, this analysis was designed as a descriptive visualization rather than a genome-wide estimate of assembly correspondence. For each matched assembler and k-mer condition, sequences < 10 kb were excluded and the 20 longest scaffolds were selected to provide sufficiently long sequence blocks for interpretable alignment visualization. The selected scaffolds were therefore not assumed to be representative of the genome-wide assembly. Sample SUC6832 was used as the reference and sample SUC8040 as the query.

Pairwise alignments were generated using NUCmer from MUMmer v3.23 [29] with --maxmatch. One-to-one alignments were retained using delta-filter -1, and SNPs and small alignment-derived InDels were extracted using show-snps -Clr. Variant densities were calculated per megabase of the corresponding SUC6832 reference assembly.

Complementary alignments were performed using minimap2 v2.17-r941 [30] with the asm10 preset and wfmash v0.24.1 [31] with a minimum alignment length of 10 kb and minimum identity of 90%. Putative structural differences of 1–100 kb were detected using Assemblytics v2.0.1 [32] with a minimum unique anchor length of 10 kb. These differences were treated as alignment-based candidates, and overall alignment relationships were visualized using Circos v0.69-8 [33]. Because only the longest scaffolds were included to maintain visual interpretability, these Circos comparisons were used solely to illustrate long-scaffold sequence relationships and were not used to infer genome-wide collinearity or genome-wide assembly reproducibility.

### 2.5. Reference-Guided Chromosome-Scale Comparison of Assemblies

Reference-guided analyses were conducted as a downstream comparison to evaluate whether the chromosome-scale relationship between samples SUC6832 and SUC8040 remained consistent across alternative reference genomes. SPAdes v4.0.0 [11] scaffolds ≥ 10 kb were retained, and exact duplicate sequences were removed, resulting in 12,758 sequences for sample SUC6832 and 18,092 sequences for sample SUC8040.

Each sample was independently scaffolded against GCA_046055825.1, GCF_024679245.1, and GCF_000951615.1 using RagTag v2.1.0 [34] with -q 30, -f 5000, --remove-small, -t 16, and -w. RagTag v2.1.0 [34] used minimap2 v2.17-r941 [30] with the asm5 preset. Because this procedure reordered and oriented the sample-derived scaffolds according to the guide genome without replacing their nucleotide sequences, the outputs were treated as reference-guided scaffold assemblies.

For each reference genome, the SUC8040 assembly was aligned to the corresponding SUC6832 assembly using minimap2 v2.17-r941 [30] with -x asm10 -t 16 -c --cs. Only chromosome-level alignments with length ≥10 kb and mapping quality ≥40 were retained. Analyses included 25 chromosomes for GCA_046055825.1 and GCF_024679245.1 and 50 haploid chromosomes for GCF_000951615.1.

Alignments between the same chromosome on the positive strand were classified as collinear, those on the negative strand as inversion candidates, and alignments between different chromosomes as translocation candidates. Query intervals aligned to multiple retained target regions were flagged as multimapping. CIGAR-derived insertion and deletion operations were summarized as alignment events and were not interpreted as independent structural-variant calls. All rearrangement and InDel results were interpreted as alignment-derived candidates rather than experimentally validated structural variants.

### 2.6. Methods for Genomic Localization of Validated SSR Markers Across Reference Genomes

To examine the chromosome-scale distribution and reference-dependent detectability of the validated SSR markers, the 20 marker sequences were aligned independently against three reference genome assemblies, GCA_046055825.1, GCF_024679245.1, and GCF_000951615.1. Each reference assembly was converted into a nucleotide BLAST database using makeblastdb, and marker sequences were searched using BLASTn v2.17.0+ [26]. For each marker–reference combination, the highest-bitscore alignment satisfying the identity and query-coverage thresholds was designated as the provisional primary genomic location, while additional high-confidence hits were retained to assess potential multi-mapping. Hits with sequence identity ≥95% and query coverage ≥90% were considered high-confidence genomic matches. Chromosome accession, alignment start and end positions, strand orientation, sequence identity, query coverage, and the number of retained hits were recorded. Markers with more than one high-confidence hit were classified as potentially multi-mapping. Chromosome lengths were obtained from the corresponding reference assembly files, and the genomic positions of the markers were visualized using chromosome ideograms. Because the reference genomes differed in chromosome number and assembly structure, genomic positions were interpreted independently for each reference rather than assumed to be directly orthologous among assemblies.

### 2.7. Pseudo-Reference Construction, Read Mapping, and Joint Variant Calling

A sample-derived pseudo-reference was constructed from the SPAdes v4.0.0 [11] default multi-k assembly of SUC6832. Scaffolds ≥ 10 kb were retained, sorted by length, and exact duplicate sequences were removed using hash-based sequence comparison. The resulting assembly was scaffolded against the chromosome-level *Pseudorasbora elongata* reference genome GCA_046055825.1 using RagTag v2.1.0 [34] with -q 30, -f 5000, --remove-small, -t 16, and -w. The 25 RagTag scaffolds assigned to the 25 guide chromosomes were identified from the scaffold identifiers and AGP annotations and extracted using SAMtools v1.23.1 faidx [28] and used as the nuclear pseudo-reference. The final pseudo-reference comprised 25 scaffolds with a total length of 161,816,264 bp.

Paired-end reads from SUC6832 and SUC8040 were quality-filtered using fastp v1.3.3 [35] with automatic adapter detection, a minimum Phred score of 20, and a minimum retained read length of 50 bp. Filtered reads from both samples were independently aligned to the SUC6832-derived pseudo-reference using BWA-MEM v0.7.17-r1188 [27] with sample-specific read-group information. Alignments were coordinate-sorted and indexed using SAMtools v1.23.1 [28]. Mapping performance was summarized using SAMtools v1.23.1 [28] flagstat, idxstats, and coverage. The overall mapping rates were 89.12% for SUC6832 and 87.84% for SUC8040.

Callable regions were defined separately for each sample using SAMtools v1.23.1 [28] depth with the -aa option. Consecutive positions with read depth ≥5 were merged into BED intervals, and the intersection between samples was retained as the common callable region. This common region comprised 368,052 intervals spanning 145,121,223 bp, corresponding to approximately 89.7% of the pseudo-reference.

Joint variant calling was performed using GATK v4.6.2.0 [36]. HaplotypeCaller was run separately for each sample in GVCF mode over intervals with pre-calling depth ≥ 5 in both samples; final genotype callability was subsequently determined after genotype-level filtering. The resulting GVCFs were imported into GenomicsDB using GenomicsDBImport and jointly genotyped with GenotypeGVCFs. Variants were hard-filtered using QD < 2.0, QUAL < 30.0, FS > 60.0, and MQ < 40.0, and only records marked PASS were retained using bcftools v1.23.1 [37]. The PASS dataset was subsequently restricted to biallelic SNPs and InDels. Variant records were retained when site-level MQ was ≥40, and individual genotypes were retained when DP was ≥5, GQ was ≥20, and heterozygous allele balance was 0.25–0.75; genotypes failing these criteria were set to missing before downstream analyses. Hard filtering retained 2,473,794 PASS records. Subsequent restriction to biallelic SNPs and InDels and application of genotype-level DP, GQ, MQ, and allele-balance filters yielded a final analysis dataset of 2,012,643 variants.

## 3. Results

### 3.1. Development of the Validated SSR Marker Set

The initial CLC assemblies of individuals SUC6832 and SUC8040 comprised 234,966 and 269,777 contigs, with total lengths of 185.37 and 236.29 Mb, respectively (Table 1). The N50 values were 786 bp for SUC6832 and 897 bp for SUC8040, and the GC contents were 39.23% and 38.88%, respectively. A total of 41,389 SSRs were identified in SUC6832 and 59,338 in SUC8040, corresponding to densities of 223.28 and 251.12 SSRs/Mb. Dinucleotide repeats were the most abundant SSR class, accounting for 78.20% and 77.15% of all SSRs, respectively.

Of the 7854 candidate SSR loci that met the sequence and primer-design criteria used for cross-sample comparison, 936 loci (11.92%) were recovered from both SUC6832 and SUC8040 (Table 2). Among these, 852 loci had identical SSR motifs, whereas 84 had different motifs. The shared loci consisted of 821 dinucleotide, 106 trinucleotide, and 9 tetranucleotide SSRs. From the shared candidate pool, 50 loci meeting the primer-design requirements were selected for experimental PCR screening. Twenty of the 50 tested loci yielded reliable amplification and polymorphic allele profiles and constituted the final validated marker panel, corresponding to a validation success rate of 40%. These 20 experimentally validated loci were subsequently used as the benchmark set for the multi-assembler comparison. Detailed characteristics of the 20 experimentally validated SSR loci, including GenBank accession numbers, primer sequences, repeat motifs, allele-size ranges, annealing temperatures, number of alleles, observed and expected heterozygosity, polymorphism information content, and Hardy–Weinberg equilibrium statistics, are provided in Supplementary Table S1.

### 3.2. Sequence Data and Multi-Assembler Assembly Characteristics

SPAdes yielded assembly sizes of 795.2 Mb for SUC6832 and 815.1 Mb for SUC8040, with N50 values of 5.5 and 7.0 kb and BUSCO completeness of 30.2% and 35.2%, respectively. Across the remaining assemblers, assembly sizes ranged from 460.2 to 870.6 Mb, N50 values from 1.1 to 2.5 kb, and BUSCO completeness from 6.4% to 16.6% (Table 3). In contrast, application of the same BUSCO workflow and actinopterygii_odb12 dataset to the chromosome-level *P. parva* and *P. elongata* reference genomes yielded complete BUSCO scores of 98.7% (7110/7207) and 99.0% (7134/7207), respectively. *P. parva* contained 98.0% single-copy and 0.6% duplicated BUSCOs, with 0.3% fragmented and 1.1% missing, whereas *P. elongata* contained 98.3% single-copy and 0.7% duplicated BUSCOs, with 0.3% fragmented and 0.7% missing (Table S3).

Wall-clock time ranged from 1.4 to 23.6 h, CPU time from 10.9 to 215.4 core-hours, peak RSS from 32.1 to 135.0 GB, and CPU utilization from 482% to 2221%. SPAdes required 2.8–3.9 h and 32.1–34.2 GB peak RSS, whereas the highest peak RSS values were observed for SOAPdenovo2 at k-mer sizes 71 and 127 in SUC8040, reaching 135.0 and 134.3 GB, respectively.

### 3.3. Recovery and Reproducibility of Target Marker Sequences

SPAdes recovered all 20 target-marker sequences at high confidence in both SUC6832 and SUC8040, with each query showing ≥95% identity and ≥90% query coverage (Table 4). All 20 SSR loci, including 100-bp flanking sequences on both sides of the repeat tract, were recovered at high confidence in both SPAdes assemblies. Independent examination of the experimentally validated primer-binding sequences showed 100% nucleotide identity for both forward and reverse primers at all 20 loci, with no mismatches detected (Supplementary Table S2). Thus, the validated marker sequences retained intact primer annealing sites in addition to satisfying the broader full-locus recovery criteria.

ABySS detected all 20 markers at all k-mer sizes. High-confidence marker recovery in SUC6832 was 20/20, 18/20, and 15/20 at k = 41, 71, and 127, respectively, whereas SUC8040 showed 19/20, 19/20, and 18/20. High-confidence 100-bp flanking-region recovery ranged from 18/20 to 20/20 in SUC6832 and was 20/20 at all k-mer sizes in SUC8040.

SOAPdenovo2 detected all 20 markers and flanking regions in both samples. High-confidence marker recovery ranged from 13/20 to 17/20 in SUC6832 and from 17/20 to 20/20 in SUC8040. High-confidence flanking-region recovery ranged from 16/20 to 18/20 in SUC6832 and from 18/20 to 20/20 in SUC8040.

MaSuRCA detected 17–19 markers in each sample. High-confidence marker recovery ranged from 9/20 to 17/20 in SUC6832 and from 14/20 to 18/20 in SUC8040. High-confidence flanking-region recovery ranged from 12/20 to 19/20 and from 16/20 to 19/20, respectively.

### 3.4. Contig-Level Comparison of Marker-Recovering Assemblies

**Figure 1:**
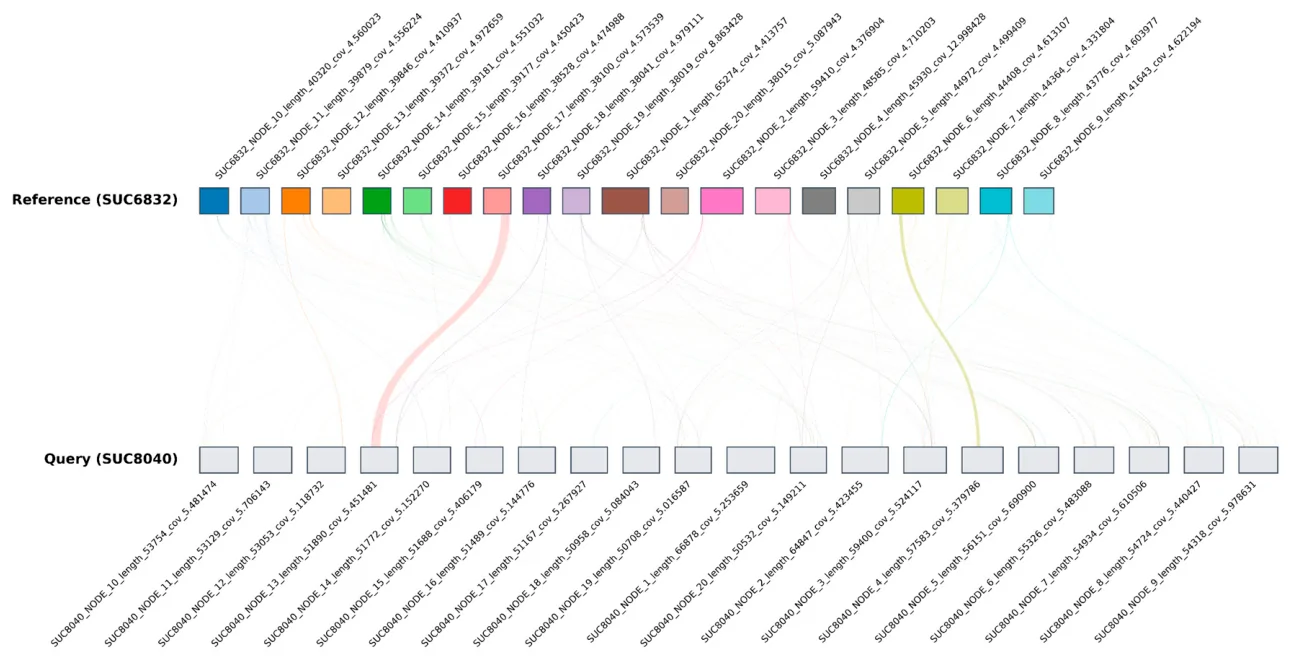
Contig-level alignment between SUC6832 and SUC8040 assemblies generated using SPAdes. The 20 longest scaffolds (>10 kb) from the SPAdes assemblies of SUC6832 and SUC8040 were compared. SUC6832 scaffolds are shown as colored reference blocks and SUC8040 scaffolds as gray query blocks. Ribbons indicate retained sequence alignments, with ribbon colors corresponding to the SUC6832 reference scaffolds. SPAdes was run using default k-mer sizes of 21, 33, 55, 77, 99, and 127.

In the descriptive visualization restricted to the 20 longest scaffolds, which were selected to provide sufficiently long sequence blocks for readable comparison rather than to represent the genome-wide assemblies, the SPAdes assemblies showed more extensive visible sequence correspondence between SUC6832 and SUC8040 than the selected configurations of the other assemblers (Figure 1). Numerous alignments connected most of the 20 longest scaffolds, including several broad ribbons representing longer retained alignment spans under the applied filtering criteria. Additional thinner links were distributed across multiple scaffolds, reflecting fragmented or multi-segment alignments. These patterns describe only the selected long-scaffold subset and should not be extrapolated to the genome-wide assemblies.

**Figure 2:**
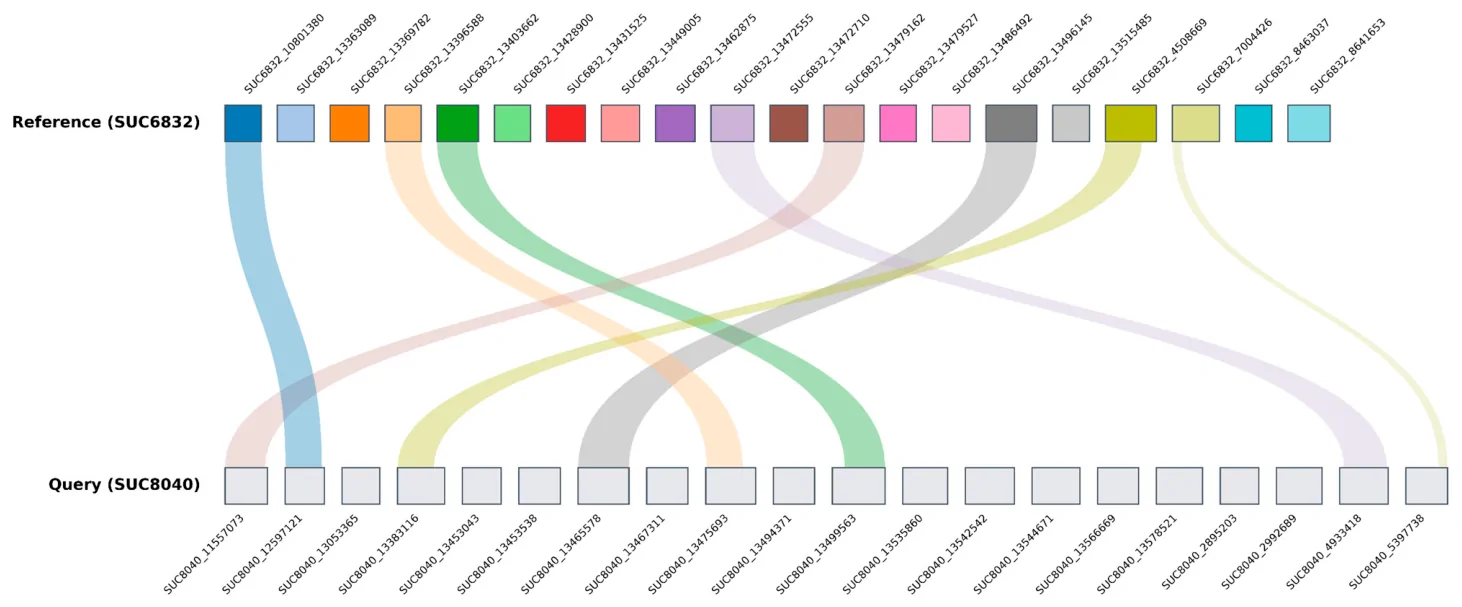
Contig-level alignment between SUC6832 and SUC8040 assemblies generated using ABySS at k-mer 41. The 20 longest scaffolds (>10 kb) from the ABySS k-mer 41 assemblies of SUC6832 and SUC8040 were compared. Colored blocks represent SUC6832 reference scaffolds, gray blocks represent SUC8040 query scaffolds, and ribbons indicate retained sequence alignments between the two assemblies.

The ABySS k-mer 41 assemblies exhibited a smaller number of dominant contig-level relationships (Figure 2). Several long ribbons connected individual SUC6832 scaffolds to SUC8040 scaffolds, whereas many of the selected scaffolds showed no visible retained alignment. Some alignments connected scaffolds occupying different positions in the two selected scaffold sets, which may reflect differences in assembly fragmentation, scaffold ranking, or sequence organization between the samples.

**Figure 3:**
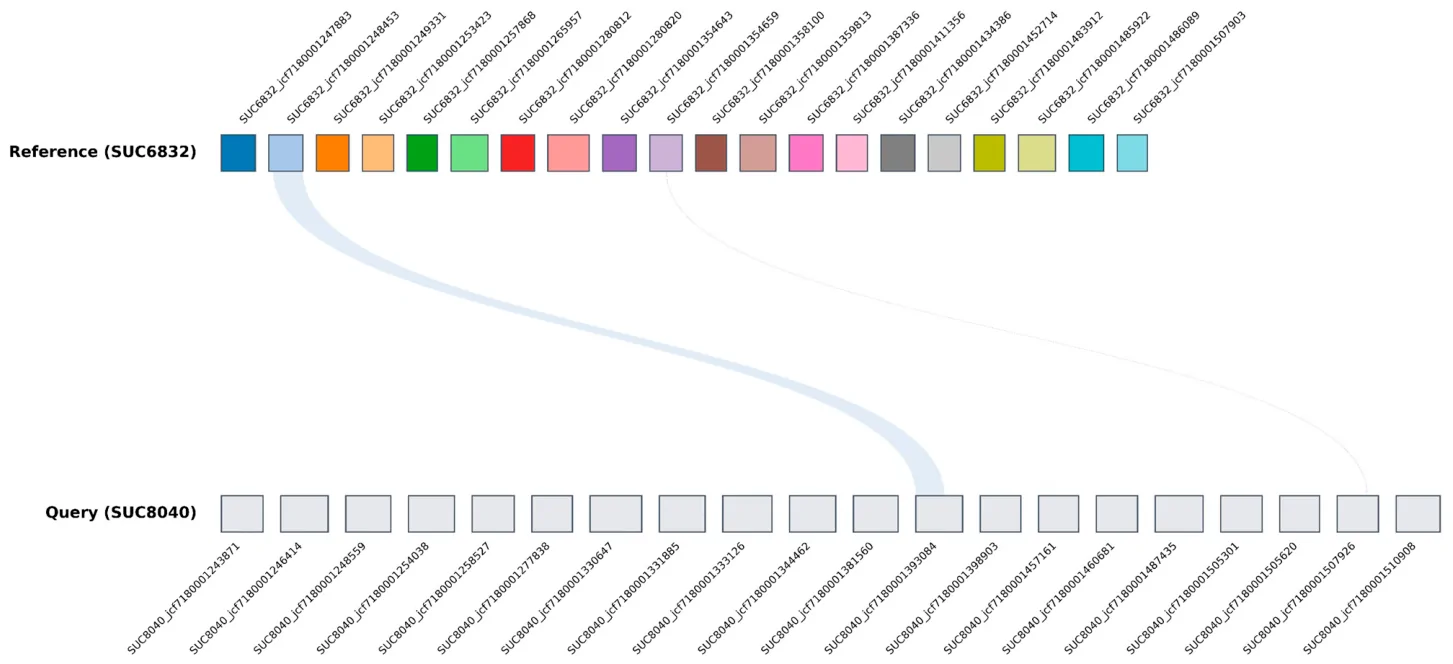
Contig-level alignment between SUC6832 and SUC8040 assemblies generated using MaSuRCA at k-mer 41. The 20 longest scaffolds (>10 kb) from the MaSuRCA k-mer 41 assemblies of SUC6832 and SUC8040 were compared. SUC6832 reference scaffolds are shown in color and SUC8040 query scaffolds in gray. Connecting ribbons represent retained alignments and illustrate the limited contig-level correspondence between the two assemblies.

Within the selected 20-longest-scaffold subset, the MaSuRCA k-mer 41 comparison showed limited visible contig-level correspondence (Figure 3). One major alignment connected a SUC6832 scaffold to a SUC8040 scaffold, while only one additional weak alignment was visible. Most of the 20 longest scaffolds lacked retained alignments between the two samples.

**Figure 4:**
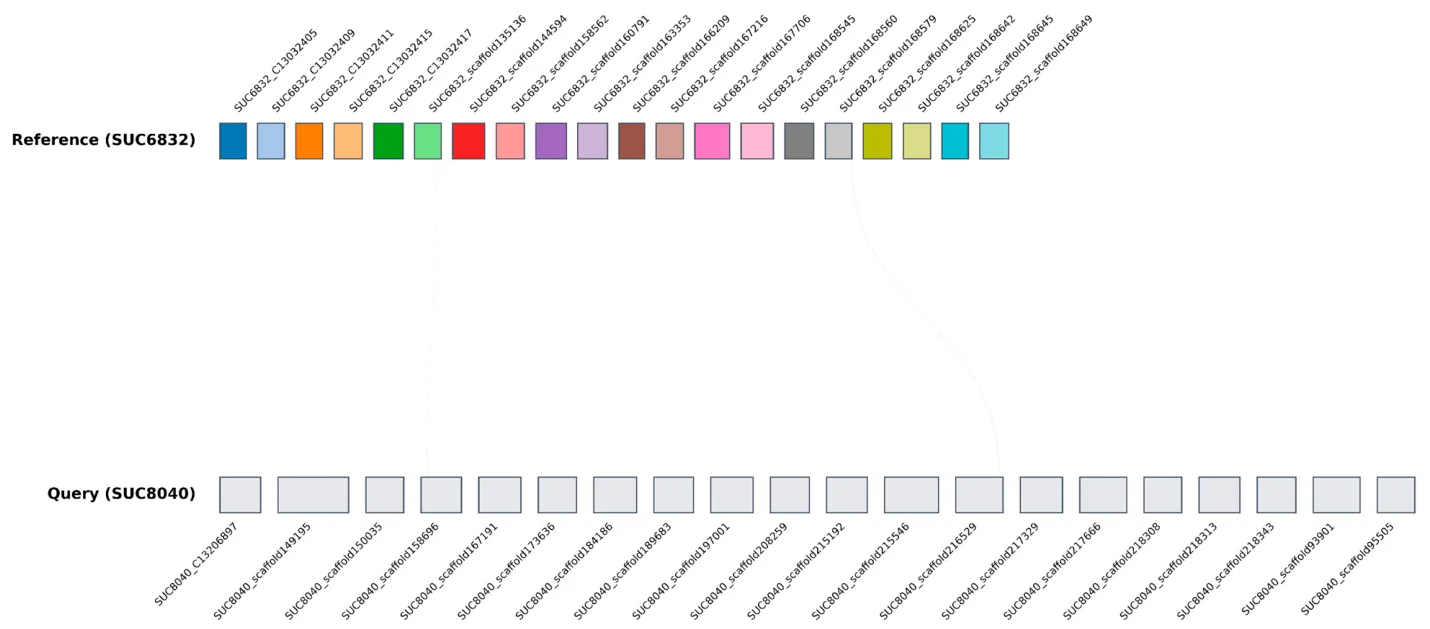
Contig-level alignment between SUC6832 and SUC8040 assemblies generated using SOAPdenovo2 at k-mer 71. The 20 longest scaffolds (>10 kb) from the SOAPdenovo2 k-mer 71 assemblies of SUC6832 and SUC8040 were compared. Colored and gray blocks represent SUC6832 reference and SUC8040 query scaffolds, respectively. Connecting ribbons indicate retained sequence alignments between SUC6832 and SUC8040 scaffolds.

Within the selected long-scaffold visualizations, the SOAPdenovo2 k-mer 71 assemblies showed the sparsest visible correspondence among the four comparisons (Figure 4). Only a small number of faint alignments were retained, and most SUC6832 and SUC8040 scaffolds were unconnected. Under the applied alignment and scaffold-selection criteria, the SOAPdenovo2 assemblies showed limited retained correspondence among the 20 longest scaffolds.

### 3.5. Reference-Guided Comparison of Chromosome-Level Scaffold Assignments Between SUC6832 and SUC8040

**Figure 5:**
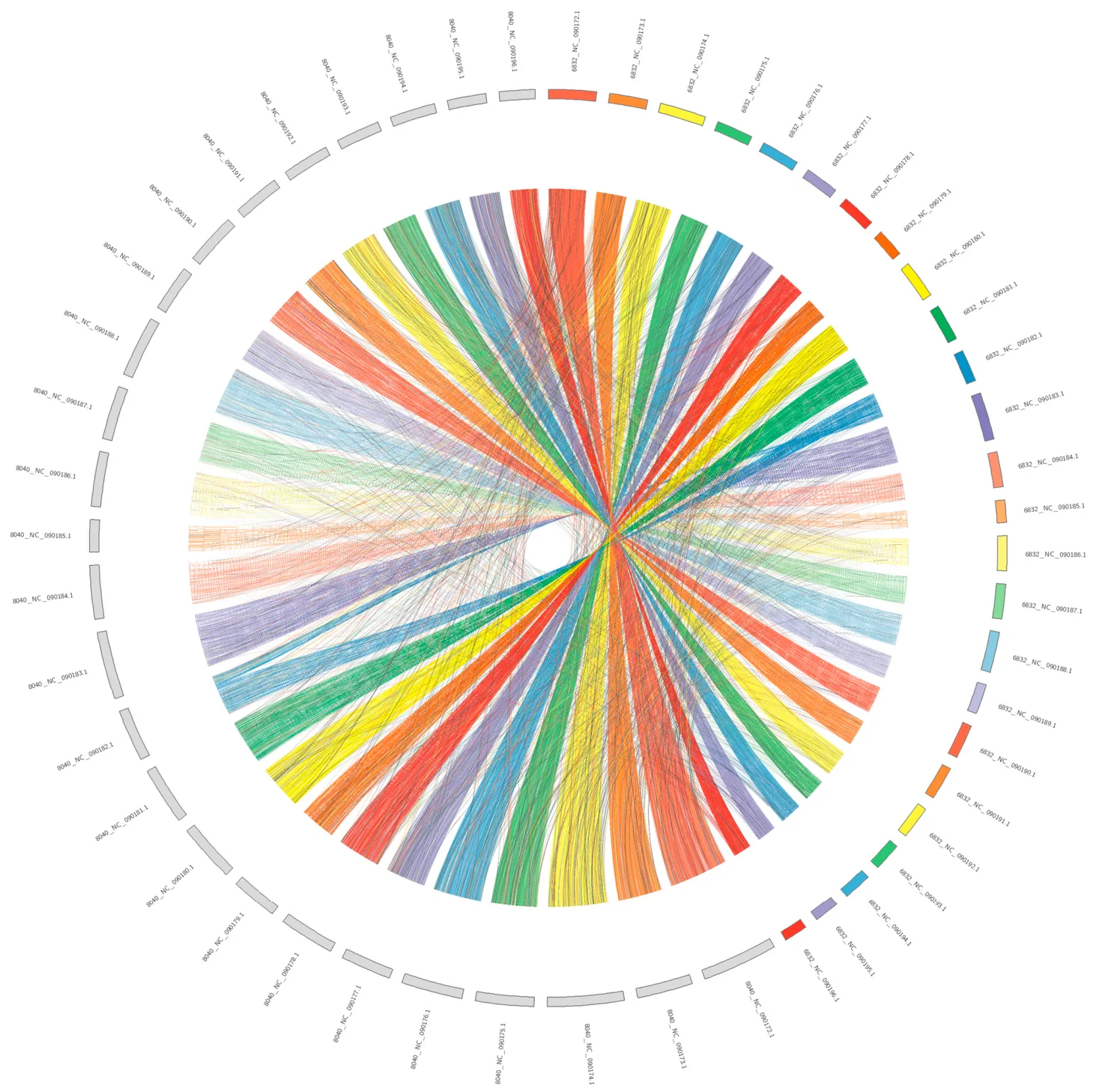
Reference-guided chromosome-scale alignment between SUC6832 and SUC8040 using the *P. elongata* genome. SUC6832 and SUC8040 assemblies were independently scaffolded against the *P. elongata* reference genome GCA_046055825.1. Colored outer segments represent SUC6832 reference-guided chromosomes, gray segments represent the corresponding SUC8040 chromosomes, and internal ribbons indicate retained alignments ≥10 kb with mapping quality ≥40. Ribbon colors correspond to the SUC6832 target chromosomes.

The *P. elongata*-guided comparison retained 4399 alignments spanning 78.35 Mb, with a mean sequence identity of 90.35% (Table 5). Because both sample assemblies had been independently ordered and oriented against the same guide genome, these alignments were interpreted only as reference-guided sequence correspondences and not as independent evidence of chromosome-scale collinearity or structural variation. Same-chromosome, same-orientation alignments accounted for 77.54 Mb (98.96%) of the retained aligned sequence. The small remaining fraction comprising opposite-orientation or cross-chromosome links was retained in Table 5 as alignment-derived discordance of undetermined biological validity. In the Circos plot, most ribbons connected corresponding chromosomes between SUC6832 and SUC8040, whereas only a small number of links crossed between noncorresponding chromosomes (Figure 5).

**Figure 6:**
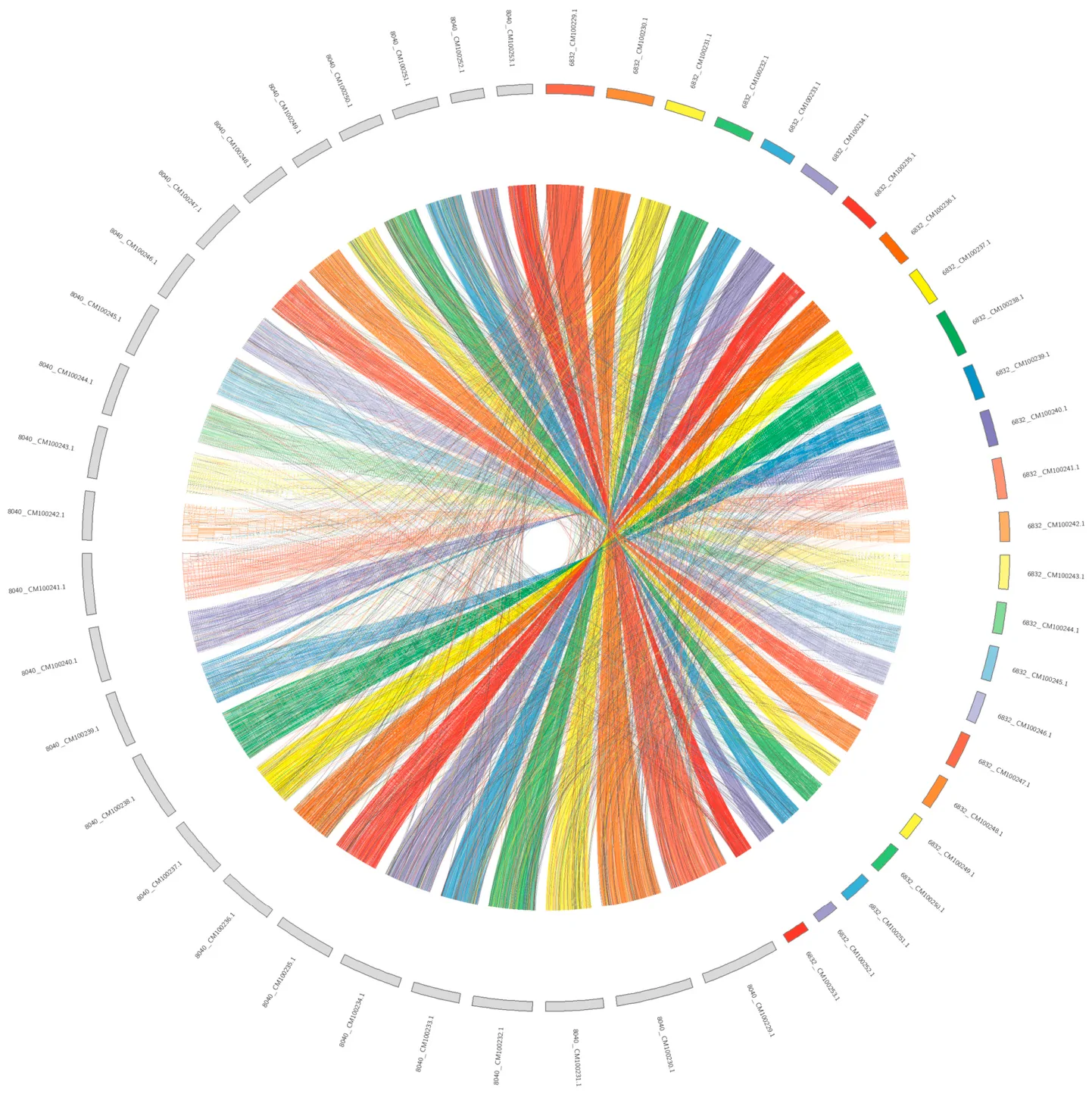
Reference-guided chromosome-scale alignment between SUC6832 and SUC8040 using the *P. parva* genome. SUC6832 and SUC8040 assemblies were independently scaffolded against the *P. parva* reference genome GCF_024679245.1. Colored outer segments indicate SUC6832 reference-guided chromosomes, gray segments indicate SUC8040 chromosomes, and internal ribbons represent retained alignments ≥ 10 kb with mapping quality ≥40. Ribbon colors are assigned according to the SUC6832 target chromosomes.

The *P. parva*-guided comparison retained 4311 alignments totaling 77.20 Mb, with a mean identity of 90.27% (Table 5). A total of 4244 collinear alignments covered 76.39 Mb, accounting for 98.95% of the aligned sequence. As in the *P. elongata*-guided analysis, the relatively small fraction of opposite-orientation or cross-chromosome links was treated only as reference-guided alignment discordance rather than evidence of biological rearrangements. The Circos plot showed a dense set of chromosome-matched links, with relatively few cross-chromosomal connections compared with the overall number of retained alignments (Figure 6).

**Figure 7:**
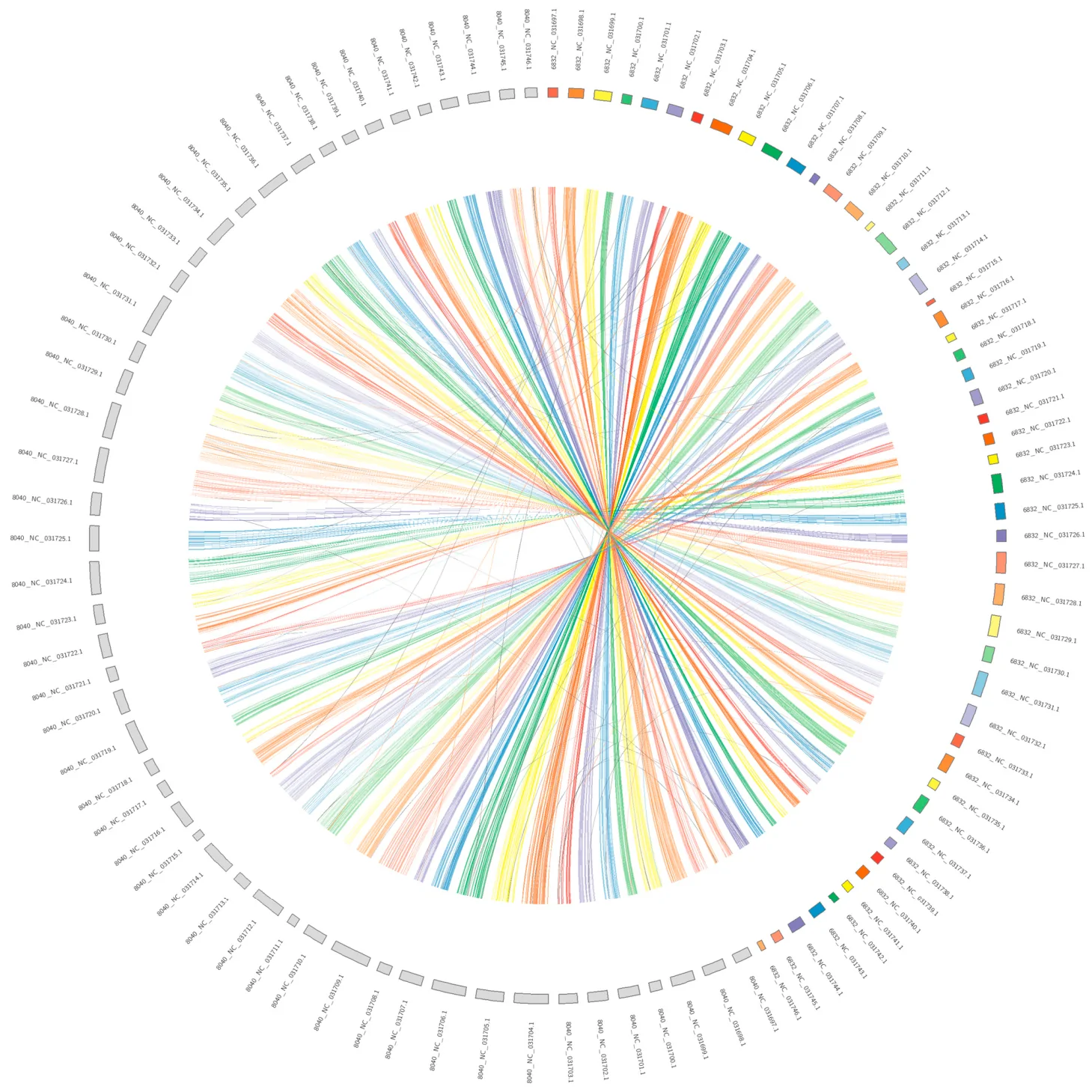
Reference-guided chromosome-scale alignment between SUC6832 and SUC8040 using the *Cyprinus carpio* genome. SUC6832 and SUC8040 assemblies were independently scaffolded against the *Cyprinus carpio* reference genome GCF_000951615.1. Colored outer segments represent SUC6832 reference-guided chromosomes, gray segments represent the corresponding SUC8040 chromosomes, and internal ribbons indicate retained alignments ≥ 10 kb with mapping quality ≥ 40. The comparison includes 50 haploid chromosomes, and ribbon colors correspond to the SUC6832 target chromosomes.

The *C. carpio*-guided comparison retained only 729 alignments spanning 13.27 Mb, substantially less than the approximately 77–78 Mb retained with either *Pseudorasbora* reference. Although the mean identity of the retained *C. carpio* alignments was slightly higher (91.37%), this reflected a much smaller subset of conserved sequence. Accordingly, the principal result of the reference-guided comparison was the substantially greater retained sequence span obtained with the more closely related *Pseudorasbora* references, rather than the number of alignment-orientation or cross-chromosome discordances. In the Circos plot, alignments were distributed across 50 chromosomes but were visibly sparser and more fragmented than in the two *Pseudorasbora*-guided comparisons (Figure 7).

### 3.6. Genomic Localization of Validated SSR Markers Across Reference Genomes

**Figure 8:**
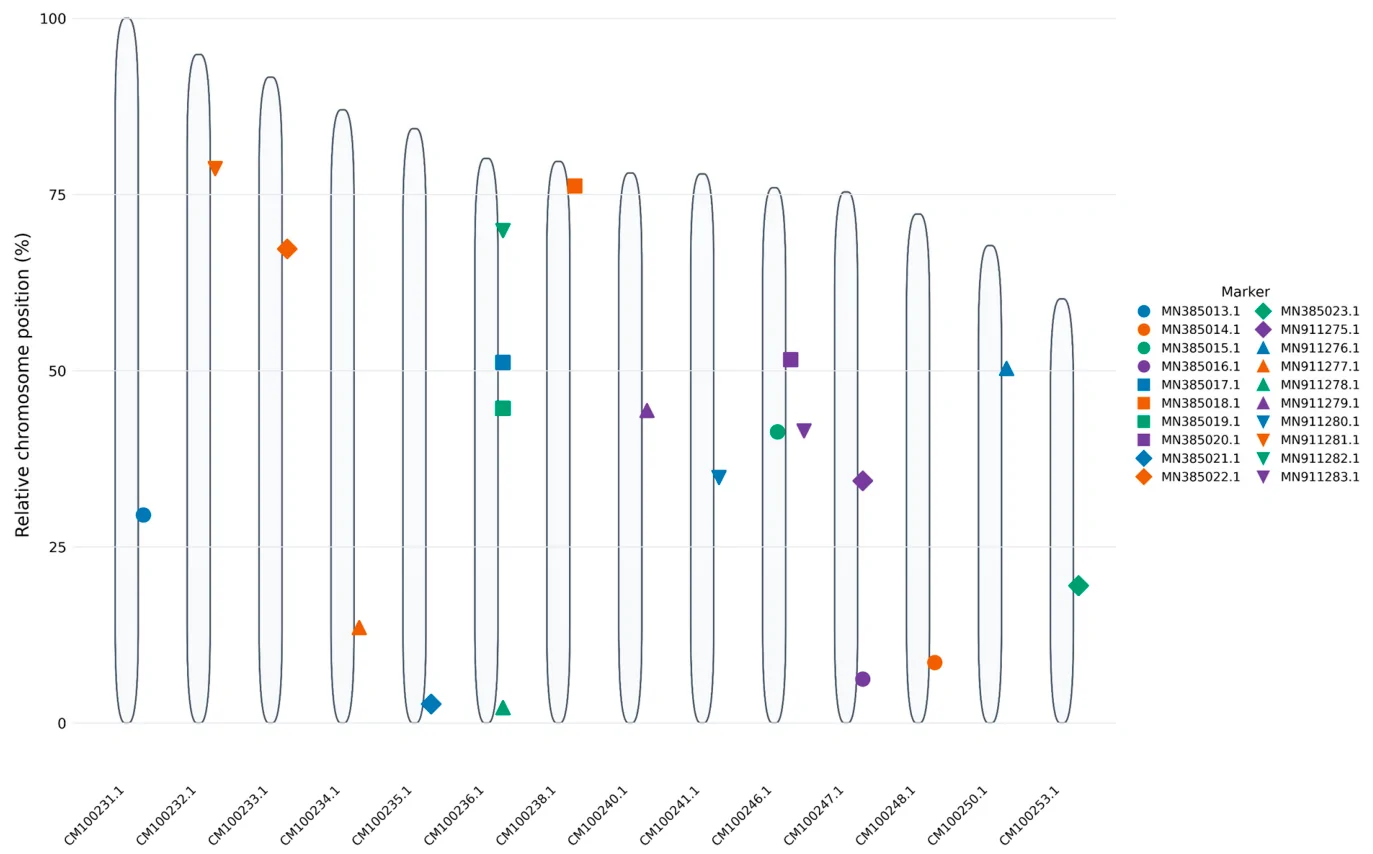
Chromosomal distribution of validated SSR markers in the *P. elongata* reference genome. Chromosomal locations of the 20 validated SSR markers in the *P. elongata* reference genome assembly GCA_046055825.1. Marker positions were determined using high-confidence sequence matches with identity ≥ 95% and query coverage ≥ 90%. Colored points and labels indicate individual SSR markers along the 25 chromosomes.

In the *Pseudorasbora elongata* reference assembly, the primary high-confidence matches of all 20 validated SSR markers were provisionally assigned to 14 chromosomes (Figure 8). Most chromosomes contained one marker, whereas several chromosomes carried two or three markers. Marker positions ranged from approximately 2% to 79% of chromosome length.

**Figure 9:**
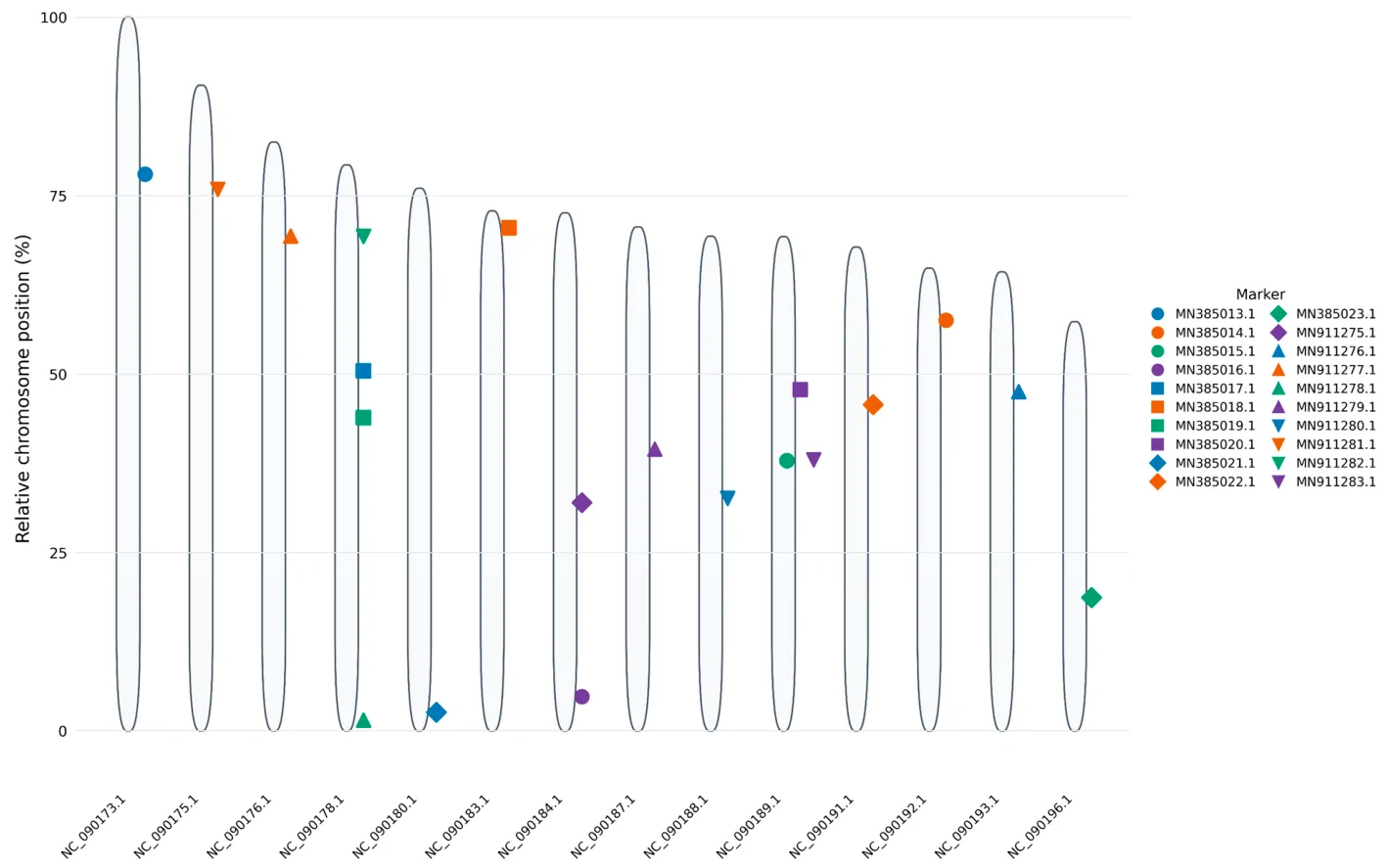
Chromosomal distribution of validated SSR markers in the *P. parva* reference genome. Chromosomal locations of the 20 validated SSR markers in the *P. parva* reference genome assembly GCF_024679245.1. High-confidence genomic positions were defined by sequence identity ≥ 95% and query coverage ≥ 90%. Colored points and labels represent individual SSR markers distributed across the 25 chromosomes.

Similarly, all 20 markers were mapped to 14 chromosomes in the *Pseudorasbora parva* reference assembly (Figure 9). Their relative positions ranged from approximately 2% to 78%, with multiple markers occurring on several chromosomes.

**Figure 10:**
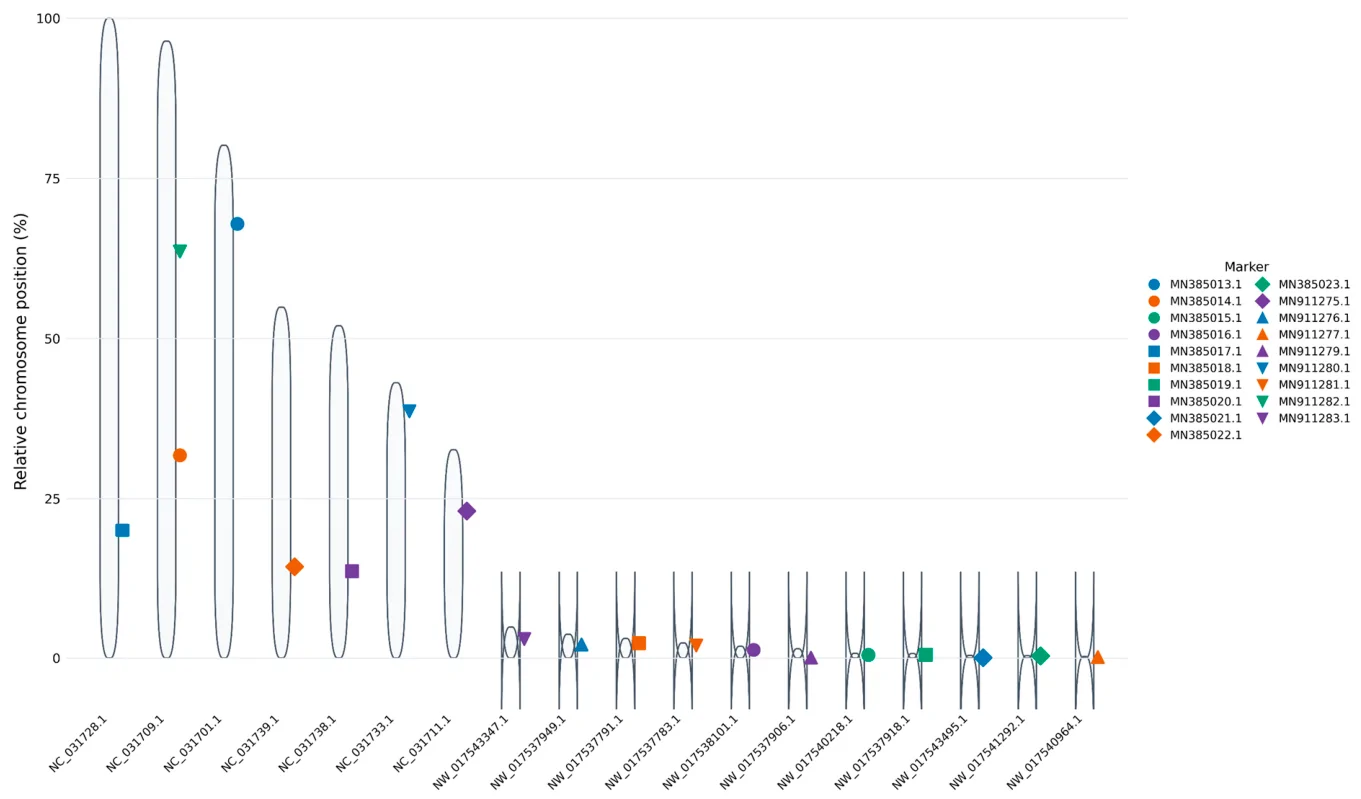
Chromosomal distribution of validated SSR markers in the *C. carpio* reference genome. Chromosomal locations of the 20 validated SSR markers in the *C. carpio* reference genome assembly GCF_000951615.1. Marker positions were assigned using high-confidence alignments with identity ≥ 95% and query coverage ≥ 90%. Colored points and labels indicate individual SSR markers across the 50 haploid chromosomes. Only chromosomes and unplaced scaffolds containing at least one mapped marker are shown.

In the *Cyprinus carpio* reference assembly, the 20 markers were distributed across both chromosomes and unplaced scaffolds (Figure 10). Eight markers were located on seven chromosomes, while the remaining 12 markers were assigned to 11 unplaced scaffolds. Chromosome-associated markers occurred at approximately 14–68% of chromosome length, whereas scaffold-associated markers were generally positioned near scaffold termini.

**Figure 11:**
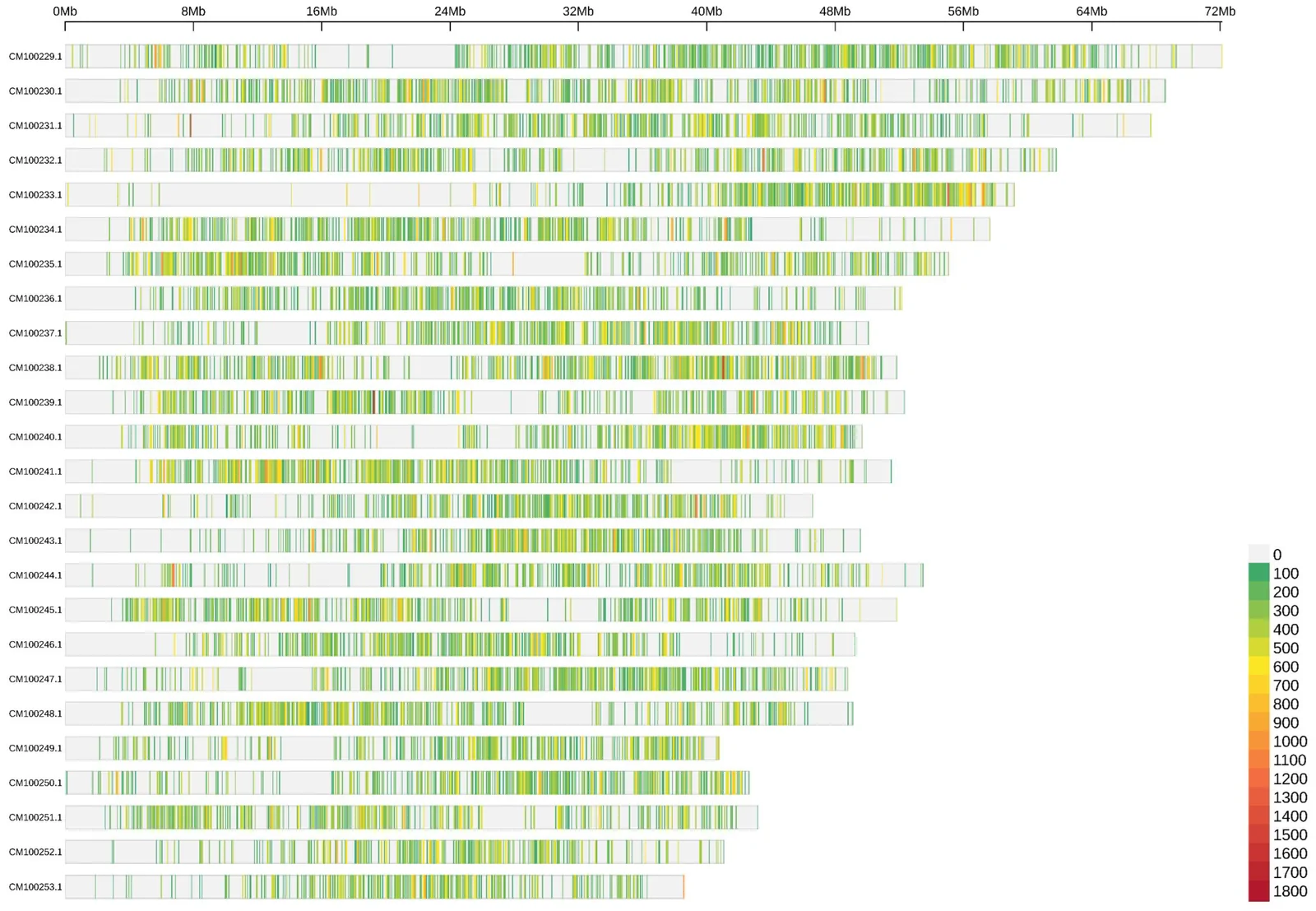
Variant density across 25 chromosomes based on the SUC6832-derived pseudo-reference genome. Jointly called variants from SUC6832 and SUC8040 were identified using the SUC6832-derived pseudo-reference scaffolded against the *P. elongata* genome. Variant coordinates were projected onto the corresponding 25 original *P. elongata* chromosomes using RagTag coordinate information. Variant density was calculated in 100-kb windows, with colors indicating the number of variants per window and gray representing windows without detected variants.

Joint variant calling against the SUC6832-derived pseudo-reference retained 2,012,643 provisional variant records after the applied site- and genotype-level filters. Variant coordinates on the SUC6832-derived pseudo-reference were projected onto the corresponding *P. elongata* guide-coordinate framework using RagTag alignment and AGP information and summarized in 100-kb windows (Figure 11). Projected variants were detected across all 25 chromosomes, with most windows containing low-to-moderate variant densities and several localized windows showing higher densities. Variant density ranged from 0 to approximately 1800 variants per 100-kb window.

After quality filtering, 2,012,643 variants were retained, comprising 1,569,461 SNPs and 443,182 InDels (Table 6). The transition and transversion counts were 810,608 and 758,853, respectively, yielding a transition-to-transversion (Ti/Tv) ratio of 1.068.

SUC6832 contained 661,952 heterozygous SNPs and 10,602 homozygous alternate genotypes, whereas SUC8040 contained 594,858 heterozygous SNPs and 424,534 homozygous alternate genotypes. Relative to the total pseudo-reference length of 161.82 Mb, heterozygous SNP densities were 4090.8 and 3676.1 per Mb for SUC6832 and SUC8040, respectively.

Among sites callable in both individuals, 341,837 shared alternate variants were identified. In addition, 198,012 SUC6832-specific and 332,013 SUC8040-specific candidate variants were detected. Of 871,862 jointly callable sites, 185,121 had the same genotype state and 686,741 had different genotype states, corresponding to concordance and discordance rates of 0.212 and 0.788, respectively.

## 4. Discussion

### 4.1. Assembly Fragmentation, Assembler Choice, and the Efficiency of SSR Marker Recovery

The initial CLC assemblies of SUC6832 and SUC8040 were highly fragmented (234,966 and 269,777 contigs; N50 = 786 and 897 bp) yet still yielded 41,389 and 59,338 SSR loci, corresponding to densities of 223.28 and 251.12 SSRs/Mb. Dinucleotide repeats dominated both datasets (78.20% and 77.15%), which is consistent with the repeat-class composition reported for other teleost SSR discovery studies; for example, dinucleotide motifs were also the most abundant class in the *Betta splendens* transcriptome-based SSR survey [38] and in genome-survey-based microsatellite characterization of the endangered cyprinid *Procypris rabaudi* [39]. The predominance of dinucleotide repeats is broadly consistent with previous teleost SSR surveys, suggesting that the fragmented assemblies retained the major repeat-class pattern. Nevertheless, only 936 of 7854 examined loci (11.92%) were shared between the two individuals, and the substantial difference in total SSR counts indicates that locus-level discovery from fragmented assemblies remained sensitive to biological sampling, sequencing depth, and assembly fragmentation. This limited cross-sample reproducibility further illustrates that SSR discovery from short-read sequence data can be influenced by dataset-specific sequencing characteristics and assembly performance [8,9].

Under the sequencing and parameter conditions examined here, SPAdes produced higher N50 and BUSCO completeness values than the tested ABySS, SOAPdenovo2, and MaSuRCA configurations, with assembly sizes of 795.2 and 815.1 Mb, N50 values of 5.5 and 7.0 kb, and BUSCO completeness values of 30.2% and 35.2% for SUC6832 and SUC8040, respectively. Across the alternative assembler configurations, N50 values ranged from 1.1 to 2.5 kb and BUSCO completeness from 6.4% to 16.6%. This ranking is broadly consistent with previous benchmarks in which SPAdes performed favorably on several real-read datasets across different coverage levels, whereas its apparent disadvantage relative to MaSuRCA was observed only on simulated data—a discrepancy the authors attributed to coverage bias [10,11,40]. The relatively low BUSCO completeness of even the SPAdes assemblies (30.2–35.2%) indicates substantial genome-wide fragmentation. Importantly, the low completeness is unlikely to be explained primarily by incompatibility with the actinopterygii_odb12 lineage dataset. When the identical BUSCO workflow was applied to the chromosome-level *P. parva* and *P. elongata* reference genomes, complete BUSCO recovery reached 98.7% and 99.0%, respectively. These results demonstrate that the selected BUSCO lineage dataset is capable of recovering nearly all expected conserved orthologs from closely related gobionine genomes. The two datasets contained approximately 11.0 and 12.2 Gb of sequence. Because an independent genome-size estimate was not available and the present sequencing depth was insufficient for a stable k-mer-based estimate, true physical genome coverage was not calculated. For descriptive purposes, dividing the raw sequence yields by the corresponding SPAdes assembly spans of 795.2 and 815.1 Mb yielded sequence-yield-to-assembly-span ratios of approximately 13.8× for SUC6832 and 14.9× for SUC8040. These values should not be interpreted as independent genome-coverage estimates. The limited amount of short-read sequence data likely constrained the absolute completeness and contiguity attainable by all assemblers, particularly in repetitive, heterozygous, or locally under-covered regions where de Bruijn graph continuity is difficult to maintain [41,42,43]. However, all assembler configurations within each individual were generated from the same underlying raw-read dataset and therefore received the same input sequence depth. Consequently, the observed differences among assembler configurations cannot be explained by differences in sequencing depth alone. Rather, they reflect how assembler-specific graph construction, k-mer strategy, error handling, and parameter implementation interacted with the same limited short-read data. Conserved genes may nevertheless remain fragmented across multiple scaffolds under these conditions and fail to satisfy the criteria for complete BUSCO recovery [44]. Thus, the markedly lower BUSCO completeness of the *P. nigra* assemblies is more consistent with the combined effects of limited short-read sequencing depth, extensive assembly fragmentation, and assembler-specific reconstruction limitations than with divergence from the actinopterygii_odb12 dataset. Because sequencing depth was held constant across assembler configurations within each individual, relative differences among assemblers reflect performance under the same input-data conditions; nevertheless, the present study cannot determine how those relative differences would change at substantially higher sequencing depth. The present analyses also do not allow the relative contributions of incomplete sequence sampling and assembly failure to be quantified independently.

Importantly, these BUSCO values indicate that the assemblies should not be regarded as complete reference-quality genomes. The contrast with the approximately 99% BUSCO completeness of the two chromosome-level *Pseudorasbora* references further emphasizes the genome-wide incompleteness of the present *P. nigra* short-read assemblies. Their intended application in this study was more restricted: reconstruction of SSR loci together with sufficient flanking sequence for marker development. In this context, the recovery of all 20 experimentally validated loci and their flanking regions in both SPAdes assemblies demonstrates that an assembly with limited genome-wide completeness can nevertheless retain high utility for a defined locus-level application. Marker recovery should therefore be interpreted as an application-specific complement to, rather than a substitute for, genome-wide completeness metrics such as BUSCO and N50.

Marker recovery followed the same ordering but was not a simple function of assembly statistics. SPAdes recovered all 20 validated markers and all 20 flanking regions at high confidence in both individuals, whereas high-confidence recovery in ABySS declined monotonically with k in SUC6832 (20/20, 18/20, and 15/20 at k = 41, 71, and 127), and MaSuRCA recovered as few as 9/20. This k-dependent loss is consistent with the trade-off in de Bruijn graph assembly, in which larger k-mer sizes may improve repeat resolution but reduce recovery in regions affected by limited local read support or sequencing error [40,45]. Recovery was generally higher in SUC8040 than in SUC6832, paralleling the larger read set and higher read-quality values of SUC8040. This pattern suggests that sequencing depth and read quality may have contributed alongside assembler-specific effects.

Taken together, these results indicate that conventional assembly metrics alone do not fully describe locus-level utility. SOAPdenovo2 required up to 135.0 GB peak RSS in SUC8040, while its high-confidence marker recovery varied among k-mer settings and samples. By comparison, SPAdes recovered all 20 markers in both samples with peak memory requirements of 32.1–34.2 GB [15]. We therefore suggest that marker-centered recovery provides an application-specific benchmark that complements conventional metrics such as N50 and BUSCO when assemblies are generated for validated genetic-marker development. Whereas N50 and BUSCO characterize genome-wide contiguity and conserved gene-space completeness, respectively, recovery of experimentally validated SSR loci directly evaluates whether sequences required for the intended marker-development application have been reconstructed.

An additional limitation is the small number of biological samples. Only two individuals were analyzed, and the present comparison should therefore be interpreted as a technical benchmark across two independent short-read datasets rather than as a population-level assessment. The use of two individuals allowed us to examine whether marker-recovery patterns were reproducible across biological samples, but it does not capture within-population variation or establish that the observed assembler ranking will remain unchanged across additional individuals, populations, sequencing depths, library preparations, or sequencing platforms. Broader benchmarking with replicated datasets will therefore be required to evaluate the generality of these results.

### 4.2. Reference-Guided Chromosome-Scale Comparison Between SUC6832 and SUC8040 and the Genomic Placement of Validated Markers

Reference-guided scaffolding produced markedly different amounts of comparable sequence depending on the reference used. The high collinear fractions should be interpreted cautiously because the SUC6832 and SUC8040 assemblies were independently ordered and oriented against the same guide genome; thus, part of the apparent chromosome-scale correspondence was imposed by the reference-guided scaffolding procedure [34,46]. Consequently, part of the apparent chromosome-scale correspondence may reflect the shared scaffolding framework rather than independently resolved chromosome-scale structure. The substantially smaller aligned span obtained with the *C. carpio* reference is consistent with an effect of greater phylogenetic distance, although its allotetraploid history, larger chromosome number, duplicated regions, and reference assembly architecture may also have contributed [47,48]. This interpretation is also consistent with RAD-seq analyses in salmonids showing that increasing reference divergence can increase missing genotype calls and bias population-genetic estimates [49]. Our results extend this pattern to Gobioninae and suggest that phylogenetically closer references are preferable for provisional chromosome-scale comparisons in this group. The slightly higher mean identity among retained *C. carpio* alignments should not be interpreted as greater overall similarity, because stringent filtering likely retained only a relatively small subset of highly conserved regions.

The rearrangement signal was small in absolute terms—6 inversion candidates (70.37 kb) and 62 translocation candidates (742.30 kb) under the *P. elongata* guide, and comparable numbers under *P. parva*—amounting to roughly 1% of aligned sequence. Because both assemblies were ordered by the same external reference, such signals cannot be interpreted as validated structural variants. Reference-guided scaffolders are explicitly known to import reference structure into the output, and although RaGOO/RagTag pseudomolecules were shown by Hi-C validation to contain few structural errors, erroneous reference bias was nonetheless detectable [34,46]. A subsequent evaluation in *Megalobrama amblycephala* reported thousands of misassemblies and 192 inter-chromosomal cross-links in uncorrected reference-guided scaffolds, of which the cross-links were eliminated only after RagTag correction [50]. Our conservative filtering (≥10 kb, MAPQ ≥ 40) and our explicit treatment of these events as alignment-derived candidates are consistent with those cautions.

Localization of the validated markers reproduced the same reference-dependence. All 20 markers were placed on 14 chromosomes in both *P. elongata* and *P. parva*, at 2–79% and 2–78% of chromosome length, whereas in *C. carpio* only 8 markers were assigned to 7 chromosomes and 12 fell on unplaced scaffolds, mostly near scaffold termini. Two factors plausibly contribute: the greater sequence divergence of the flanking regions, and the allotetraploid history of *C. carpio*, whose 50 haploid chromosomes and retained duplicates make unique placement of short marker sequences intrinsically harder [51,52]. This parallels reports that heterospecific references lose mapping coverage disproportionately in repeat-rich windows [48].

From a marker-development perspective, the recovery of all 20 loci on 14 of 25 chromosomes in the two relatively close *Pseudorasbora* references indicates broad but non-uniform genome-wide distribution, with several chromosomes carrying two or three markers. Comparable clustering has been described when microsatellite panels developed for population genetics are retrospectively anchored to chromosome-level assemblies in other cyprinids [53]. Because the three references differ in chromosome number and assembly architecture, we interpret marker coordinates independently for each reference rather than as orthologous positions, and the *Pseudorasbora*-based coordinates provide a more plausible provisional framework for approximate marker localization, although linkage and genome-scan applications will require validation with a species-specific chromosome-level assembly or genetic map.

### 4.3. Pseudo-Reference-Based Variant Analysis and Comparison with Previous Population-Genetic Studies

Based on the total 161.82-Mb pseudo-reference length, the observed heterozygous SNP counts corresponded to 4090.8 and 3676.1 heterozygous SNP calls per Mb for SUC6832 and SUC8040, respectively; these values were descriptive densities rather than estimates of genome-wide heterozygosity. These values approximate the proportion of heterozygous SNP positions within the analyzed callable sequence but should not be interpreted as genome-wide heterozygosity because repetitive, unplaced, and insufficiently covered regions were excluded. These estimates fall within the range reported from genome surveys of other cyprinids, including 0.44% in the endangered *Procypris rabaudi* [39] and 0.53% in *Percocypris pingi* [54], and below the 0.64–1.00% reported for the highly heterozygous marine teleost *Nemadactylus macropterus* [55]. The observed heterozygous SNP densities are broadly compatible with previous microsatellite evidence that some genetic variation remains within *P. nigra*, although direct comparison between SNP-based sequence heterozygosity and microsatellite *H*o should be made cautiously [2].

The most conspicuous result was the asymmetry between individuals: SUC6832 carried only 10,602 homozygous alternate genotypes, compared with 424,534 in SUC8040, and the proportion of matching genotype states across 871,862 variant sites with non-missing filtered genotypes in both individuals was 0.212. Part of this asymmetry is a direct consequence of reference bias, because the pseudo-reference was built from SUC6832 and its own alleles are therefore preferentially called as homozygous reference; overestimation of divergence and distortion of heterozygosity when reads are mapped to a non-matching reference are well documented [48,56]. The present analysis cannot determine how much of this asymmetry reflects biological divergence rather than reference bias, individual variation, or differences in genotype callability. SUC6832 originates from the Geumgang system (Yudeungcheon) and SUC8040 from the Mangyeonggang, and both microsatellite (*F*_ST_ = 0.135–0.168, *K* = 2 with MG separated) and mitochondrial *cytb* analyses have independently identified the Mangyeonggang population as the most strongly differentiated and genetically distinct unit within the species [1,2]. The 332,013 variant sites carrying an alternate genotype only in SUC8040 may reflect individual genetic differences and technical asymmetry arising from the SUC6832-derived pseudo-reference; population- or drainage-level contributions cannot be evaluated with one individual per drainage. Because only one individual per drainage was analyzed, these variants cannot be considered population- or drainage-specific.

Projected variant densities ranged from 0 to approximately 1800 per 100-kb window across all 25 *P. elongata*-derived chromosomes, with most windows at low-to-moderate density and a minority of sharply elevated windows. Such localized excesses may arise from collapsed duplications, repetitive regions, or local mapping ambiguity; genuine concentrations of sequence variation cannot be distinguished from these technical explanations without additional validation [57].

The low Ti/Tv ratio of 1.068 indicates that the filtered variant set likely retains a substantial contribution from mismapping, duplicated or repetitive sequences, or other false-positive calls, although the expected species-specific Ti/Tv ratio is unknown. The variant set should therefore be treated as exploratory until it is evaluated using reciprocal pseudo-references, stricter mappability filters, and additional individuals.

More broadly, the distribution of filtered variant records across all 25 reference-guided scaffolds demonstrates that a sample-derived pseudo-reference can support genome-wide variant calling in a technical sense, although the accuracy and population-genetic utility of the resulting calls require additional validation. Previous studies have similarly used de novo pseudo-references to align reads, identify SNPs, and infer population structure in non-model organisms. For example, Boehm et al. constructed a pseudo-reference from the individual with the greatest sequencing depth and used it to resolve regional population structure in the lined seahorse *Hippocampus erectus*, whereas Chen et al. demonstrated that a pseudo-reference enabled reliable SNP genotyping and linkage analysis in *Brassica napus* despite the absence of a species-level reference genome [58,59]. Comparative analyses of RAD-seq workflows have further shown that de novo or pseudo-reference-based approaches can support standard population-genomic analyses, although estimates of heterozygosity, demographic history, and allele frequencies may remain sensitive to assembly, mapping, and filtering choices [60]. Accordingly, the SUC6832-derived pseudo-reference should not be regarded as equivalent to a complete *P. nigra* reference genome, particularly for repetitive regions, structural variation, or chromosome-specific functional interpretation. Nevertheless, after stringent genotype filtering, it may provide a provisional resource for identifying candidate genome-wide variation and prioritizing loci for subsequent population-level conservation-genomic studies until a high-quality species-specific reference genome becomes available.

Collectively, under the sequencing platform, read depth, parameter settings, and benchmarking criteria examined here, SPAdes showed the most favorable observed combination of validated SSR recovery, N50, BUSCO completeness, runtime, and peak memory use among the tested configurations. Reference-guided organization of the SPAdes assembly further enabled approximate chromosome-scale marker localization and exploratory variant discovery, although these pseudo-reference-based results remain sensitive to reference bias, assembly fragmentation, and the limited number of analyzed individuals.

From a conservation perspective, the experimentally validated SSR loci and their provisional genomic placements provide a practical molecular resource for future genetic monitoring of *P. nigra*, including assessments of genetic diversity and differentiation among remnant and restored populations. Nevertheless, the present study has several important limitations, including the use of only two biological individuals, modest short-read sequencing depth, substantial genome-wide assembly fragmentation, and dependence on heterospecific guide genomes and a sample-derived pseudo-reference. Future work should therefore expand sampling across major drainage systems, develop a chromosome-level *P. nigra* reference genome using long-read and chromosome-conformation sequencing, and validate candidate genomic variants and marker positions in larger population samples. Such efforts will be required before the exploratory genomic results presented here can be translated into robust population-genomic inference and conservation-management applications.

## 5. Conclusions

In the two low-coverage Illumina short-read datasets examined, assembler choice and k-mer configuration were associated with substantial differences in the recovery of PCR-validated microsatellite loci. Under these specific sequencing conditions, SPAdes showed the highest validated-marker recovery, N50, and BUSCO completeness among the tested configurations, together with relatively low peak memory use. These findings are specific to the read depth, read characteristics, and assembler settings evaluated here and should not be extrapolated directly to high-depth short-read datasets or long-read genome assemblies. Reference-guided organization of the SPAdes assembly enabled provisional guide-coordinate marker placement and exploratory variant calling, but these results remain sensitive to reference bias, assembly fragmentation, and the analysis of only two individuals. Marker-centered benchmarking therefore provides a useful complement to conventional assembly statistics when assemblies are generated primarily for molecular-marker development.

## Figures and Tables

**Table 1:** Assembly statistics and SSR characteristics of two individuals.

Sample	No. of Contigs	Total Assembly Length (bp)	N50 (bp)	Longest Contig (bp)	GC Content (%)	Total SSRs	SSR-Containing Contigs	Compound SSRs
SUC6832	234,966	185,366,397	786	9591	39.23	41,389	36,647	1243
SUC8040	269,777	236,292,781	897	10,970	38.88	59,338	51,094	2050

**Table 2:** Shared SSR markers between individuals SUC6832 and SUC8040.

Comparison	Shared SSR Markers	Shared Markers (%)	Identical SSR Motifs	Different SSR Motifs
6832 vs. 8040	936	11.92	852	84

**Table 3:** Performance comparison of de novo genome assemblies generated using four short-read assemblers and different k-mer settings for samples SUC6832 and SUC8040.

Assembler	K-mer	Sample	Assembly Size (Mb)	Contigs ≥ 1 kb	Largest Contig (kb)	N50 (kb)	BUSCO Complete (%)	Clock Time (hr)	CPU Utilization (%)	Peak RSS (GB)
SPAdes	21-33-55-77-99-127	SUC6832	795.2	175,846	65.3	5.5	30.2	2.8	660.0	32.1
ABySS	127		773.5	291,852	17.1	1.5	9.7	23.6	873.0	63.3
ABySS	41		460.2	186,900	16.7	1.4	10.1	7.9	2048.0	53.5
ABySS	71		632.6	243,634	24.9	1.7	12.4	8.7	2163.0	66.3
MaSuRCA	127		525.6	227,048	23.6	1.6	7.6	5.6	2125.0	66.1
MaSuRCA	41		500.6	203,919	18.3	1.8	10.9	10.1	1802.0	65.6
MaSuRCA	71		560.2	225,934	23.6	1.9	10.8	7.2	2137.0	66.6
SOAPdenovo2	127		781.0	279,185	13.3	1.1	6.4	2.1	529.0	133.9
SOAPdenovo2	41		504.3	192,216	15.5	1.3	10.1	2.4	1018.0	72.2
SOAPdenovo2	71		682.6	246,225	17.7	1.3	10.4	3.3	778.0	71.1
SPAdes	21-33-55-77-99-127	SUC8040	815.1	155,525	66.9	7.0	35.2	3.9	482.0	34.2
ABySS	127		837.7	295,752	21.7	2.0	14.6	12.0	1647.0	65.8
ABySS	41		477.5	194,635	17.2	1.5	11.7	9.0	2059.0	53.8
ABySS	71		663.5	245,741	23.9	2.0	15.9	9.7	2221.0	66.6
MaSuRCA	127		670.1	259,119	28.3	2.3	12.4	9.4	1802.0	68.3
MaSuRCA	41		599.1	224,640	22.9	2.3	14.8	5.4	2084.0	67.2
MaSuRCA	71		679.3	243,356	31.5	2.5	16.6	8.4	2014.0	68.4
SOAPdenovo2	127		870.6	311,415	27.1	1.4	9.9	1.5	869.0	134.3
SOAPdenovo2	41		555.5	210,943	27.8	1.5	11.9	1.6	897.0	72.5
SOAPdenovo2	71		736.6	255,271	35.9	1.6	13.8	1.4	778.0	135.0

Assembly performance was evaluated using total assembly size, number of contigs ≥ 1 kb, largest contig length, contig N50, BUSCO completeness, elapsed clock time, mean CPU utilization, and peak resident set size (RSS). SPAdes was run using multiple k-mer sizes (21, 33, 55, 77, 99, and 127), whereas ABySS, MaSuRCA, and SOAPdenovo2 were independently evaluated at k-mer sizes of 41, 71, and 127. Clock time represents the total elapsed runtime, CPU utilization indicates the average utilization relative to a single CPU core, and peak RSS represents the maximum physical memory usage during assembly.

**Table 4:** Recovery and high-confidence validation of previously developed SSR markers and their flanking regions across de novo assemblies generated using different assemblers and k-mer settings.

Assembler	Sample	K-mer	Marker Detected (%)	Marker High Confidence (%)	SSR Flanking Region Detected (100 bp)	SSR Flanking Region High Confidence (%)
SPAdes	SUC6832	21-33-55-77-99-127	100.0	100.0	100	100
ABySS		41	100.0	100.0	100	100
ABySS		71	100.0	90.0	100	95
ABySS		127	100.0	75.0	100	90
SOAPdenovo2		41	100.0	85.0	100	85
SOAPdenovo2		71	100.0	85.0	100	90
SOAPdenovo2		127	100.0	65.0	100	80
MaSuRCA		41	95.0	85.0	95	95
MaSuRCA		71	85.0	75.0	80	80
MaSuRCA		127	90.0	45.0	85	60
SPAdes	SUC8040	21-33-55-77-99-127	100.0	100.0	100	100
ABySS		41	100.0	95.0	100	100
ABySS		71	100.0	95.0	100	100
ABySS		127	100.0	90.0	100	100
SOAPdenovo2		41	100.0	90.0	100	90
SOAPdenovo2		71	100.0	100.0	100	100
SOAPdenovo2		127	100.0	85.0	100	100
MaSuRCA		41	95.0	90.0	95	95
MaSuRCA		71	85.0	80.0	85	85
MaSuRCA		127	90.0	70.0	90	80

A total of 20 previously developed SSR markers were evaluated in assemblies of samples SUC6832 and SUC8040. Marker detection was defined by the presence of a BLAST hit, whereas high-confidence recovery was defined as a match with ≥95% sequence identity and ≥90% query coverage. SSR flanking-region recovery was evaluated using 100-bp sequences adjacent to each SSR locus. All values are expressed as percentages based on the 20 experimentally validated SSR loci. Marker detection was defined as the presence of at least one BLAST hit, whereas high-confidence (HC) recovery required ≥95% sequence identity and ≥90% query coverage. Flanking-region recovery was assessed using 100-bp sequences adjacent to the SSR loci. Multi-k represents k-mer sizes of 21, 33, 55, 77, 99, and 127.

**Table 5:** Summary of reference-guided chromosome-scale alignments between the SUC6832 and SUC8040 assemblies.

Item	GCA_046055825.1	GCF_024679245.1	GCF_000951615.1
Scientific name	*Pseudorasbora elongata*	*Pseudorasbora parva*	*Cyprinus carpio*
Target chr SUC6832	25	25	50
Query chr SUC8040	25	25	50
Filtered alignments	4399	4311	729
Filtered alignments (bp)	78,350,364	77,201,602	13,274,989
Mean identity (%)	90.35	90.27	91.37
Collinear	4331	4244	696
Collinear (bp)	77,537,699	76,392,262	12,817,632
Inversion	6	5	1
Inversion (bp)	70,368	61,160	17,046
Translocation	62	62	32
Translocation (bp)	742,297	748,180	440,311
CIGAR available alignments	4399	4311	729
Insertion events	47,577	46,820	7555
Insertion (bp)	5,269,128	5,184,328	768,064
Deletion events	46,549	45,810	7341
Deletion (bp)	2,057,608	2,097,459	336,176

SUC6832 and SUC8040 assemblies were independently scaffolded against three reference genomes and subsequently compared at the chromosome scale. Only alignments ≥ 10 kb with mapping quality ≥ 40 were retained. Collinear alignments indicate matches between the same chromosomes in the same orientation, inversion candidates indicate matches between the same chromosomes in the opposite orientation, and translocation candidates indicate matches between different chromosomes. Insertion and deletion events were summarized from CIGAR operations of retained alignments. All rearrangements and InDels represent alignment-derived candidates rather than experimentally validated structural variants.

**Table 6:** Summary of joint variant filtering and genotype comparisons between SUC6832 and SUC8040.

Section	Metric	SUC6832	SUC8040	Both Samples	Value
VCF quality control	GATK raw joint	-	-	-	2,635,695
	GATK filtered tags	-	-	-	2,635,695
	FILTER PASS	-	-	-	2,473,794
	PASS biallelic SNP before genotype filters	-	-	-	1,839,039
	PASS biallelic InDel before genotype filters	-	-	-	541,491
	final SNP after DP GQ MQ AB filters	-	-	-	1,569,461
	final InDel after DP GQ MQ AB filters	-	-	-	443,182
	final total after DP GQ MQ AB filters	-	-	-	2,012,643
Basic variant statistics	Final biallelic SNP count	-	-	-	1,569,461
	Final biallelic InDel count	-	-	-	443,182
	Final SNP plus InDel count	-	-	-	2,012,643
	Transition SNP count	-	-	-	810,608
	Transversion SNP count	-	-	-	758,853
	Ti/Tv ratio	-	-	-	1.068
	PASS records excluded as non-biallelic SNP/InDel or other	-	-	-	93,264
	Records excluded because all sample genotypes failed DP/GQ/AB filters	-	-	-	367,887
Per individual genotype statistics	Callable genotype count	1,360,772	1,523,733	-	-
	Callable SNP genotype count	1,066,610	1,195,621	-	-
	Callable InDel genotype count	294,162	328,112	-	-
	Heterozygous genotype count	827,539	758,775	-	-
	Heterozygous SNP count	661,952	594,858	-	-
	Homozygous alternate genotype count	10,602	424,534	-	-
	Homozygous alternate SNP count	10,015	344,755	-	-
	Homozygous alternate InDel count	587	79,779	-	-
	Alternate genotype count	838,141	1,183,309	-	-
	Heterozygous SNPs per Mb reference	4090.763	3676.132	-	-
Two individual comparison	Shared alternate variants	-	-	341,837	-
	SUC6832-specific candidate alternate variants	198,012	-	-	-
	SUC8040-specific candidate alternate variants	-	332,013	-	-
Pairwise genotype-state comparison	Sites callable in both individuals	-	-	871,862	-
	Same genotype state	-	-	185,121	-
	Different genotype state	-	-	686,741	-
	Different homozygous genotype positions	-	-	85,751	-
	Shared homozygous alternate positions	-	-	6367	-
	Genotype concordance rate	-	-	0.212	-
	Genotype discordance rate	-	-	0.788	-
	Sites not callable in both individuals	-	-	1,140,781	-

## Data Availability

Microsatellite markers were deposited in Kim et al. [2]. The raw sequencing data are available in the NCBI SRA under BioProject PRJNA1502383 (accessions SRR39838245–SRR39838246). The assemblies, variant datasets, scripts, and related files are available in Zenodo at https://doi.org/10.5281/zenodo.21553177.

## Supplementary Materials

The following supporting information can be downloaded at: https://www.mdpi.com/article/10.3390/biology15201889/s1, Table S1, Characteristics of the 20 experimentally validated SSR loci of Pseudopungtungia nigra; Table S2, Primer-binding-site sequence validation for the 20 experimentally validated SSR loci; Table S3, BUSCO assessment of the chromosome-level Pseudorasbora reference genomes using the actinopterygii_odb12 lineage dataset.

## Abbreviations

SSR, simple sequence repeat; PCR, polymerase chain reaction; WGS, whole-genome sequencing; BUSCO, Benchmarking Universal Single-Copy Orthologs; QUAST, Quality Assessment Tool for Genome Assemblies; BLAST, Basic Local Alignment Search Tool; BWA, Burrows–Wheeler Aligner; CPU, central processing unit; RSS, resident set size; MAPQ, mapping quality; GATK, Genome Analysis Toolkit; GVCF, genomic variant call format; SNP, single-nucleotide polymorphism; InDel, insertion/deletion; DP, read depth; GQ, genotype quality; MQ, mapping quality; AB, allele balance; Ti/Tv, transition/transversion ratio.
