# Supplementary material for: Marker-Centered Evaluation of De Novo Assemblers Using Validated Microsatellite Loci in the Endangered Korean Fish Pseudopungtungia nigra
Source: Biology (Basel). 2026 Oct 20;15(20):1889. doi: 10.3390/biology15201889 (PMC13701378; doi:10.3390/biology15201889)
Supplement: Supplementary file 1 [file biology-15-01889-s001.zip › biology-4495032-supplementary.pdf]

Table S1. Characteristics of 20 microsatellite markers for *Pseudopungtungia nigra*

Locus	GenBank accession no.	Primer sequence (5′–3′)	Repeat motif	Size range (bp)	Ta (℃)	Na	Ho	He	PIC	PHWE
PN4	MN385013	F: TCGGTGGATGCGAGGAATAC R: TTCCGCCTGTCAGTCAAGAC	$(AC)_6$	194-202	58	4	0.700	0.585	0.506	0.376
PN23	MN385014	F: GGCACTCAAGGATAATCTGAAC R: TGTATCCGGCCTCTGTGTAG	$(GT)_{10}$	200-212	58	5	0.567	0.565	0.520	0.568
PN28	MN911275	F: CTCTCCAAATATAGTGCCATCCA R: ATCCTCCTTTCACCCTCCGT	$(AC)_{10}$	237-247	58	5	0.600	0.575	0.479	0.382
PN64	MN911276	F: TCAACACAACGGACAGATTCG R: CTATAAGTACTGCCCCGTGCT	$(AC)_8$	249-255	58	3	0.500	0.603	0.518	0.534
PN70	MN911277	F: GTCGCCAACAGCTCATCTTG R: GCTGAGAGCTTGAGTCATCAC	$(GT)_9$	268-278	58	6	0.700	0.724	0.668	0.759
PN71	MN911278	F: TCCACACCGCACCACAATG R: CGCATCACACACACCGTAAC	$(TG)_9$	214-226	58	7	0.800	0.789	0.744	0.992
PN75	MN911279	F: CCTGCATCCATGCCGTATAG R: GCTGTTATAGCGCTGATGATAG	$(AC)_9$	199-217	58	8	0.867	0.813	0.772	0.863
PN88	MN385015	F: CAACAGGCTCCACGATTGC R: CTGCCCTCGGAAATAAGATGG	$(AC)_7$	176-186	58	6	0.633	0.676	0.626	0.229
PN92	MN385016	F: CCGTGCTCATATACAGTCCTC R: CCGCATTGTTCCTCCGATTG	$(CA)_{10}$	258-266	58	5	0.600	0.710	0.653	0.009
PN94	MN385017	F: CAGCACTTCCTGTTGGAGGT R: GGAGTATGTAAGGGGTCCAGA	$(CT)_8$	100-104	58	3	0.467	0.493	0.382	0.183
PN98	MN385018	F: CAGGATGAGTCCATCGTCTC R: GCTCAGAAGTGACCGACAGA	$(GT)_{10}$	281-301	58	9	0.900	0.866	0.835	0.717
PN99	MN385019	F: AGAGGAGGTCAGATGCTATGC	$(TC)_8$	225-245	58	5	0.667	0.679	0.617	0.928

		R: GCCAATAATCCGTGCCGATAG								
PN123	MN385020	F: GGGACACACTTAGCAAGCCT<br>R: GCCAGTGAGATTGAAAGACCA	$(GT)_{10}$	213-223	58	6	0.700	0.742	0.681	0.568
PN124	MN385021	F: GAGACGCACGACTGATGAAG<br>R: GGCTAACAGGGCGATTGATTG	$(AC)_{10}$	246-258	58	6	0.733	0.728	0.675	0.264
PN125	MN385022	F: CTGCACTCACTTCCATAGACG<br>R: AACAGTGACTCAGTGGACAGG	$(TG)_{9}$	166-184	58	6	0.600	0.611	0.549	0.192
PN126	MN385023	F: CCACACTACTGAGACTAAACTG<br>R: TGACAGACCATCTTGCATTCTG	$(GT)_{9}$	180-186	58	4	0.767	0.706	0.633	0.520
PN133	MN911280	F: AGACAGCTCAGGACCAATCC<br>R: CCACTAACTGACGTGCAGAAT	$(AC)_{9}$	226-234	58	4	0.467	0.541	0.422	0.516
PN139	MN911281	F: GTGCGAGTGAACGACTGATG<br>R: ACTTGTGCCTGCTGCTAGAG	$(TG)_{8}$	208-218	58	4	0.567	0.607	0.520	0.060
PN147	MN911282	F: GGCTTATGTGGCTTCGGATAC<br>R: AGGTGAGCCTGAGAGAGAAG	$(AC)_{9}$	122-128	58	4	0.633	0.729	0.667	0.307
PN154	MN911283	F: CCCACCAGATTGTATCGGGAT<br>R: TCTGTGTTAACGGTGCAGGAA	$(TC)_{10}$	161-169	58	5	0.767	0.703	0.648	0.915

Ta annealing temperature, *Na* number of alleles, *Ho* observed heterozygosity, *He* expected heterozygosity, *PIC* polymorphic information content, *PHWE* probability of Hardy–Weinberg equilibrium

Table S2. Primer-binding-site sequence validation for the 20 experimentally validated microsatellite loci of *Pseudopungtungia nigra*.

Marker accession no.	Locus	Repeat motif	Primer direction	Primer sequence	Primer length	Marker length	Primer start positon	Primer end positon	P identity	Mismatch sequence
MN385013.1	PN4	(AC)6	F	TCGGTGGATGCGAGGAATAC	20	560	244	263	100	0
MN385013.1	PN4	(AC)6	R	TTCCGCCTGTCAGTCAAGAC	20	560	419	438	100	0
MN385014.1	PN23	(GT)10	F	GGCACTCAAGGATAATCTGAAC	22	550	150	171	100	0
MN385014.1	PN23	(GT)10	R	TGTATCCGGCCTCTGTGTAG	20	550	331	350	100	0
MN911275.1	PN28	(AC)10	F	CTCTCCAAATATAGTGCCATCCA	23	510	93	115	100	0
MN911275.1	PN28	(AC)10	R	ATCCTCCTTTCACCCTCCGT	20	510	318	337	100	0
MN911276.1	PN64	(AC)8	F	TCAACACAACGGACAGATTCG	21	508	189	209	100	0
MN911276.1	PN64	(AC)8	R	CTATAAGTACTGCCCCGTGCT	21	508	423	443	100	0
MN911277.1	PN70	(GT)9	F	GTCGCCAACAGCTCATCTTG	20	506	60	79	100	0
MN911277.1	PN70	(GT)9	R	GCTGAGAGCTTGAGTCATCAC	21	506	315	335	100	0
MN911278.1	PN71	(TG)9	F	TCCACACCGCACCACAATG	19	506	94	112	100	0
MN911278.1	PN71	(TG)9	R	CGCATCACACACACCGTAAC	20	506	287	306	100	0
MN911279.1	PN75	(AC)9	F	CCTGCATCCATGCCGTATAG	20	507	132	151	100	0
MN911279.1	PN75	(AC)9	R	GCTGTTATAGCGCTGATGATAG	22	507	313	334	100	0
MN385015.1	PN88	(AC)7	F	CAACAGGCTCCACGATTGC	19	550	153	171	100	0
MN385015.1	PN88	(AC)7	R	CTGCCCTCGGAAATAAGATGG	21	550	312	332	100	0
MN385016.1	PN92	(CA)10	F	CCGTGCTCATATACAGTCCTC	21	550	246	266	100	0
MN385016.1	PN92	(CA)10	R	CCGCATTGTTCCTCCGATTG	20	550	490	509	100	0
MN385017.1	PN94	(CT)8	F	CAGCACTTCCTGTTGGAGGT	20	550	234	253	100	0
MN385017.1	PN94	(CT)8	R	GGAGTATGTAAGGGGTCCAGA	21	550	315	335	100	0
MN385018.1	PN98	(GT)10	F	CAGGATGAGTCCATCGTCTC	20	550	190	209	100	0

MN385018.1	PN98	(GT)10	R	GCTCAGAAGTGACCGACAGA	20	550	486	505	100	0
MN385019.1	PN99	(TC)8	F	AGAGGAGGTCAGATGCTATGC	21	550	184	204	100	0
MN385019.1	PN99	(TC)8	R	GCCAATAATCCGTGCCGATAG	21	550	388	408	100	0
MN385020.1	PN123	(GT)10	F	GGGACACACTTAGCAAGCCT	20	550	179	198	100	0
MN385020.1	PN123	(GT)10	R	GCCAGTGAGATTGAAAGACCA	21	550	373	393	100	0
MN385021.1	PN124	(AC)10	F	GAGACGCACGACTGATGAAG	20	547	7	26	100	0
MN385021.1	PN124	(AC)10	R	GGCTAACAGGGCGATTGATTG	21	547	238	258	100	0
MN385022.1	PN125	(TG)9	F	CTGCACTCACTTCCATAGACG	21	500	236	256	100	0
MN385022.1	PN125	(TG)9	R	AACAGTGACTCAGTGGACAGG	21	500	388	408	100	0
MN385023.1	PN126	(GT)9	F	CCACACTACTGAGACTAAACTG	22	550	304	325	100	0
MN385023.1	PN126	(GT)9	R	TGACAGACCATCTTGCATTCTG	22	550	463	484	100	0
MN911280.1	PN133	(AC)9	F	AGACAGCTCAGGACCAATCC	20	509	117	136	100	0
MN911280.1	PN133	(AC)9	R	CCACTAACTGACGTGCAGAAT	21	509	331	351	100	0
MN911281.1	PN139	(TG)9	F	GTGCGAGTGAACGACTGATG	20	508	165	184	100	0
MN911281.1	PN139	(TG)9	R	ACTTGTGCCTGCTGCTAGAG	20	508	359	378	100	0
MN911282.1	PN147	(AC)9	F	GGCTTATGTGGCTTCCGGATAC	22	510	140	154	100	0
MN911282.1	PN147	(AC)9	R	AGGTGAGCCTGAGAGAAG	18	510	254	269	100	0
MN911283.1	PN154	(TC)10	F	CCCACCAGATTGTATCGGGAT	21	507	187	207	100	0
MN911283.1	PN154	(TC)10	R	TCTGTGTTAACGGTGCAGGAA	21	507	328	348	100	0

Primer identity represents the nucleotide identity between each experimentally validated primer sequence and its corresponding binding region within the marker sequence. Mismatch indicates the number of nucleotide differences observed within the primer-binding region.

Table S3. BUSCO assessment of the chromosome-level *Pseudorasbora* reference genomes using the actinopterygii_odb12 lineage dataset.

Metric	*P. parva* GCF_024679245.1	*P. elongata* GCA_046055825.1
Complete BUSCOs	98.7% (7,110)	99.0% (7,134)
Single-copy	98.0% (7,066)	98.3% (7,082)
Duplicated	0.6% (44)	0.7% (52)
Fragmented	0.3% (21)	0.3% (19)
Missing	1.1% (76)	0.7% (54)
Error rate	6.40%	5.30%
Internal stop codons	454	381
